# Differential involvement of cortical and cerebellar areas using dominant and nondominant hands: An FMRI study

**DOI:** 10.1002/hbm.22997

**Published:** 2015-09-29

**Authors:** Adnan A.S. Alahmadi, Matteo Pardini, Rebecca S. Samson, Egidio D'Angelo, Karl J. Friston, Ahmed T. Toosy, Claudia A.M. Gandini Wheeler‐Kingshott

**Affiliations:** ^1^ Department of Diagnostic Radiology Faculty of Applied Medical Science King Abdulaziz University (KAU) Jeddah Saudi Arabia; ^2^ NMR Research Unit Department of Neuroinflammation Queen Square MS Centre University College London (UCL), Institute of Neurology London United Kingdom; ^3^ Department of Neurosciences Rehabilitation Ophthalmology Genetics and Maternal and Child Health, University of Genoa Genoa Italy; ^4^ Department of Brain and Behavioral Sciences University of Pavia Pavia Italy; ^5^ Brain Connectivity Center C. Mondino National Neurological Institute Pavia Italy; ^6^ Wellcome Centre for Imaging Neuroscience, UCL Institute of Neurology, University College London London United Kingdom; ^7^ NMR Research Unit Department of Brain Repair and Rehabilitation Queen Square MS Centre UCL Institute of Neurology London United Kingdom; ^8^ Brain MRI 3T Mondino Research Center C. Mondino National Neurological Institute Pavia Italy

**Keywords:** FMRI, dominant, force, contra‐lateral, squeeze‐ball

## Abstract

Motor fMRI studies, comparing dominant (DH) and nondominant (NDH) hand activations have reported mixed findings, especially for the extent of ipsilateral (IL) activations and their relationship with task complexity. To date, no study has directly compared DH and NDH activations using an event‐related visually guided dynamic power‐grip paradigm with parametric (three) forces (GF) in healthy right‐handed subjects. We implemented a hierarchical statistical approach aimed to: (i) identify the main effect networks engaged when using either hand; (ii) characterise DH/NDH responses at different GFs; (iii) assess contralateral (CL)/IL‐specific and hemisphere‐specific activations. Beyond confirming previously reported results, this study demonstrated that increasing GF has an effect on motor response that is contextualised also by the use of DH or NDH. Linear analysis revealed increased activations in sensorimotor areas, with additional increased recruitments of subcortical and cerebellar areas when using the NDH. When looking at CL/IL‐specific activations, CL sensorimotor areas and IL cerebellum were activated with both hands. When performing the task with the NDH, several areas were also recruited including the CL cerebellum. Finally, there were hand‐side‐independent activations of nonmotor‐specific areas in the right and left hemispheres, with the right hemisphere being involved more extensively in sensori‐motor integration through associative areas while the left hemisphere showing greater activation at higher GF. This study shows that the functional networks subtending DH/NDH power‐grip visuomotor functions are qualitatively and quantitatively distinct and this should be taken into consideration when performing fMRI studies, particularly when planning interventions in patients with specific impairments. *Hum Brain Mapp 36:5079–5100, 2015*. © **2015 Wiley Periodicals, Inc**.

## INTRODUCTION

The neural representation of hand movements is complex. Hand movements have been reliably associated with activations of the contralateral (CL) primary motor cortex, which in turn is modulated during movement by a widespread yet only partially understood network of CL and ipsilateral (IL) cortical areas—as well as by deep grey matter and cerebellar structures. Indeed, the functional roles of the aforementioned IL and CL activated regions during motor tasks, as well as their modulation by task context (and by inter‐individual differences in handedness) represents one of the least understood facets of the functional architecture of the motor system.

Previous studies, mostly using functional magnetic resonance imaging (fMRI), of motor network activity during motor tasks performed using the dominant (DH) and nondominant hand (NDH) have reported mixed results including: CL activations—especially localized in the primary and secondary motor cortex—produced by movement of the dominant (DH) or nondominant (NDH) hands [Verstynen et al., 2005; Ward and Frackowiak 2003]; bilateral activations using either hand [Laxmi, 1998; Seong‐Gi et al., 1993; Volkmann et al., 1998]; or strong activations ipsilateral to the hand moved [Kawashima, 1993; Seong‐Gi et al., 1993; Verstynen et al., 2005]. This heterogeneity of results is difficult to interpret, especially given methodological differences between different studies. For example, some studies have focused on performing a motor task using finger tapping [Jäncke et al., 1998; Verstynen et al., 2005], repetitive [Kuhtz‐Buschbeck et al., 2008] or static [Keisker et al., 2010] power grip tasks. However, there are differences in the networks involved in controlling these movements [Keisker et al., 2010; Khorrami et al., 2011; King et al., 2014]. Additionally, task context has been variably defined by different authors, usually by appealing to the notion of “task complexity.” Several groups, for example, consider that more complex (difficult) tasks are implicitly performed with the NDH compared to the DH [Ng et al., 2008; Verstynen et al., 2005]. Other groups instead associate “complexity” with the increasing demand needed to perform the motor task [e.g., Keisker et al., 2009; Verstynen et al., 2005]. In the study presented here, we will equate complexity with the computational complexity of completing the task that can depend upon a number of factors [i.e., the complexity of the task identified with reaching each grip force (GF) level and the complexity of executing the task identified with the use of the DH versus NDH, in the ecological context of a power grip dynamic task that involves processing visual and sensory inputs from the whole hand, mimicking everyday life].

Moreover, the lateralization of neural activations during complex motor tasks performed with DH or NDH remains poorly characterized—especially when using an event‐related dynamic power grip task, which, to our knowledge, has not yet been investigated using fMRI. Characterizing neuronal responses when performing tasks with the DH or NDH is interesting, not only from a neuroscience point of view but also because neurological and neurodegenerative diseases may have selective effects on these responses. Previous fMRI studies of the visuomotor network have assumed that DH and NDH activations mirror each other, such that flipping the activations found using the NDH correspond to DH activations [e.g., Ward and Frackowiak, 2003; Ward et al., 2004, 2006, 2007].

In addition to putative asymmetries, one might ask how the response depends on the complexity of the task and how this depends on the complexity of executing it using the DH or NDH. In this study, in an attempt to contribute to this discussion, an event‐related dynamic power grip fMRI paradigm was designed with three different GF levels to experimentally manipulate complexity, while using either the DH or NDH to perform the task. The acquired data was then analysed with a succession of statistical tests purposely devised to address to the best of our knowledge the following key questions:

1. What are the functional networks engaged by the main effect of whole‐hand visually guided sensorimotor processing (irrespective of the applied forces) using the DH or NDH?
2. What are the effects of different GF levels on these functional responses?
3. Which regions show common activations in the right and left hemispheres independently of the hand used (hemisphere‐specific) and which show common CL or IL (laterality‐specific) activations, independently of the hand used?

## METHODS

### Participants

Fourteen [5F, 9M; mean age 31.0 (±4.48) years] right‐handed healthy volunteers were recruited for this study. The handedness of each subject was evaluated using the Edinburgh handedness‐scaling questionnaire [Oldfield, 1971]. The mean laterality index using the handedness questionnaire was 93 (±9). No subject had a history of neurological or psychiatric disease. The local research and ethics committee approved the study and all participants gave written informed consent.

### Paradigm

Subjects performed a power grip (repetitive dynamic squeezing) task with an MR‐compatible squeezeball using both hands unimanually. The squeezeball, used also in a previous study [Alahmadi et al., 2015], is a pneumatic flexible pad. Compression of the ball results in an air pressure measurement proportional to the force exerted, which was sampled at a rate of 20 Hz.

Two consecutive fMRI scanning sessions were performed, comprising 75 three second trials, started using a visual cue and interspersed with 75 null events (baseline). The 75 active trials were divided equally into sets of 25 trials at GF levels of 20, 40, and 60% of the subject's maximum voluntary contraction (MVC). Four inter‐trial intervals were used: 2, 4, 7.5, and 9 s. All trials were presented in a randomized and counter‐balanced order, optimised using the OptSeq software (http://www.surfer.nmr.mgh.harvard.edu/optseq *)*. Before the fMRI session, subjects were trained using a similar but different 2‐min design with GF levels ranging from 10 to 70% of their MVC. The training session consisted of: observing the task being performed and performing the task while lying in the scanner bore. Half of the subjects started the fMRI session using their right (dominant) hand, the other half started with their left hand. The visual cue was a black static horizontal bar (presented for 3 s), indicating the target level to reach. This cue was projected onto an MR‐compatible white screen and show together with a coloured bar indicating the actual force level reached, thereby providing real‐time feedback of the subject's performance.

### MRI Acquisition

A 3T Philips Achieva MR scanner (Philips Healthcare, Best, The Netherlands) with a 32‐channel head coil was used to perform a 3DT1‐weighted anatomical scan and two T2*‐weighted EPI fMRI scans. The 3DT1‐weighted sequence acquisition parameters were as follows: 3D inversion‐recovery prepared gradient‐echo (fast field echo) sequence with inversion time (TI) = 824 ms, echo time (TE)/repetition time (TR) = 3.1/6.9 ms, flip angle = 8° and voxel size = 1 mm isotropic. The fMRI sequence acquisition parameters were: TR/TE = 2,500/35 ms, 46 2.7 mm slices positioned to include the cerebellum, with 3 mm^2^ in‐plane resolution, inter‐slice gap of 0.3 mm, FOV = 192 mm^2^, SENSE factor = 2, flip angle = 90° and 200 repeated volumes.

### Data Preprocessing

Image analysis was performed using SPM8 (http://www.fil.ion.ucl.ac.uk/spm), implemented in Matlab12b (Mathworks, Sheborn, MA), using conventional preprocessing steps: slice timing, realignment, coregistration, normalization [using a symmetrical MNI template (Fonov et al., 2011)] and smoothing with an 8‐mm isotropic Gaussian kernel.

### Statistical Analysis

The statistical analysis was specifically designed to exploit available SPM8 tools such as the general linear model statistics and conjunction analysis first for a qualitative assessment followed by quantitative comparisons to answer specific questions. Details of the analysis are given here below.

#### Within‐subject

A general linear model (GLM) was constructed by defining the peak of the plateau of trial responses at the 3 GF levels as separate conditions (regressors). These covariates were convolved with a canonical hemodynamic response function and used in a conventional whole brain (statistical parametric mapping) analysis [Friston et al., 1995, 1998]. The movement parameters from the realignment step were also included in the GLM as regressors of no interest [Friston et al., 1996].

For the DH and NDH, *t*‐statistic contrasts were used at within subject level to generate contrast images (or summary statistics) for:

- Main effect of movement versus baseline (rest), independently of GF;
- Main effect of movement versus rest at each GF level;
- Positive linear effects of different GF levels on BOLD signal.

To compare CL and IL activations (see below), the main effect contrast images for each subject—and for the DH and NDH at each GF level—were flipped about the mid‐sagittal line and resliced with respect to the original (unflipped) images to generate flipped contrast images (fDH and fNDH) [Callaert et al., 2011; Van Impe et al., 2009; Ward et al., 2006, 2007]. This effectively introduces a further design factor; namely, laterality [Salmond et al., 2000]. This analysis shows true bilateral (as opposed to unilateral) activations when voxels overlap using the conjunction statistical analyses reported below. This analysis reaffirmed lateralization (or true unilateral activations) identified using the lateralization method also described in detail below.

#### Between‐subjects

The contrast images from the “within‐subject” analysis were entered into random effects analyses (RFXs), as detailed below (main effect of movement, main effect of GF, linear response analysis, and laterality). For all tests, the significance level was set at a corrected *P* < 0.05 (FWE) at cluster level with a minimum extent of 10 voxels (classes were defined using an uncorrected threshold of *P* < 0.0001; *T* = 5.11).

Conjunction analysis (CA) was performed to define common areas that were specific to the right and left hemisphere responses as well as areas that were common to CL and IL activations, independently of using the DH or NDH.

Anatomical designation of the regional effects were based upon maxima or peaks in the ensuing statistical parametric maps: cluster peaks were first extracted using the Peak_nii software (http://www.nitrc.org/projects/peak_nii) and then labelled using the cytoarchitectonic probability maps provided by the SPM Anatomy toolbox [Eickhoff et al., 2005, 2007b]. The SPM anatomy toolbox included individual areas such as: primary motor cortex [Brodmann areas (BA) 4a and 4p] [Geyer et al., 1996], premotor cortex (BA 6) [Geyer, 2003], primary somatosensory cortex (BA 3a, 3b, 1) [Geyer et al., 1996], somatosensory cortex (BA 2) [Grefkes et al., 2001], parietal operculum or S2 (OP 1‐4) [Eickhoff et al., 2006a, 2006b,2007a], intraparietal sulcus [Choi et al., 2006; Scheperjans et al., 2008a, 2008b], superior parietal cortex [Scheperjans et al., 2008a,,b], inferior parietal cortex [Caspers et al., 2008], and visual areas (V4) [Rottschy et al., 2007] (V4) [Malikovic et al., 2007]. Some Figures were generated using FIVE (http://www.nmr.mgh.harvard.edu/harvardagingbrain/People/AaronSchultz/FIVE) and MRIcron (http://www.nitrc.org/projects/mricron).

In addition, in order to identify parietal and cerebellar regions, activations obtained at whole brain level as described below in the various sections were mapped to specific templates:

#### Parietal and premotor regions

The corresponding anatomical areas were extracted from the SPM anatomy toolbox and masked with the significant activations. Moreover, in order to investigate in more detail the premotor subdivisions, the dorsal premotor cortex (PMd) and the ventral premotor cortex (PMv) were identified using the human motor area template (HMAT) (Mayka et al., 2006). Therefore, we used the resultant maps for the premotor and parietal cortices to represent significant activations within key regions of these two cortical regions.

#### Cerebellar regions

**Figure 1:**
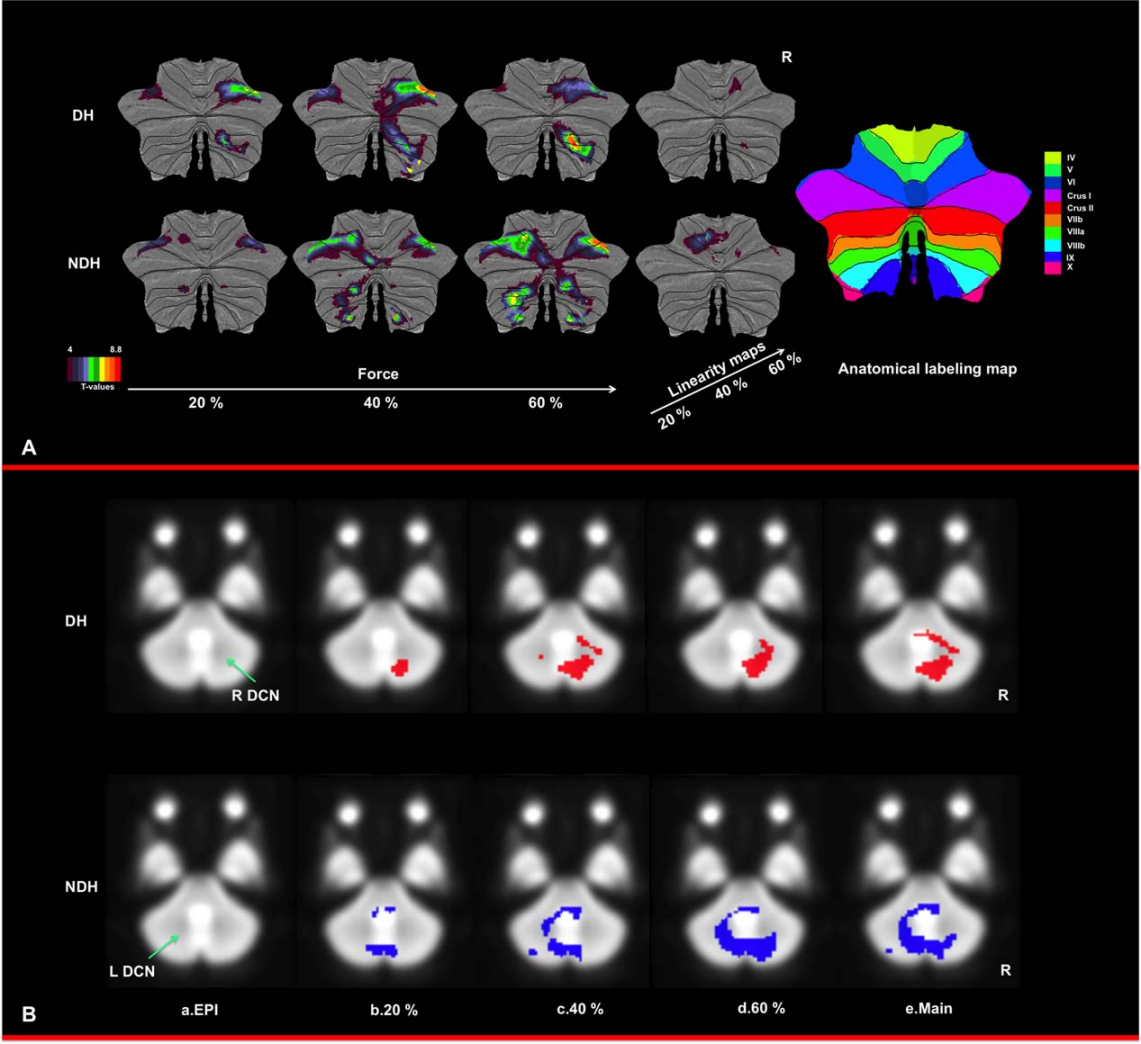
(**A**) Illustration of activation maps for DH and NDH on the SUIT flattened cerebellum. Clearly, the anterior and superior posterior ipsilateral cerebellum are involved at each GF. The contralateral cerebellum, on the other hand, is highly involved when using the NDH. (**B**) Illustration of the involvement of the deep cerebellar nuclei with the task. (a) The dentate cerebellar nuclei (DCN) is the largest nucleus and has a high iron content, therefore appearing hypointense on the EPI template, at either side of the cerebellar midline (green arrow). (b–d) Activations produced by either the DH (top row, red) or NDH (bottom row, blue), at GF levels of b. 20%, c. 40%, d. 60% of maximum voluntary contraction (MVC), (e) Main effect of grip, showing that as the GF increases the DCNs are more engaged, hence indicating an interaction with complexity of both the task and its execution. [Color figure can be viewed in the online issue, which is available at http://wileyonlinelibrary.com.]

The spatially unbiased infratentorial flattened template (SUIT) for the cerebellum [Diedrichsen, 2006] was used together with the Caret Human Connectome workbench (http://brainmap.wustl.edu/caret) [Van Essen et al., 2002] to map the activated volumes to the SUIT flattened map (Diedrichsen, J. & Zotow, E. (submitted); http://www.icn.ucl.ac.uk/motorcontrol/imaging/suit_flatmap.htm). In addition, the overlap between the cerebellar activations and the deep cerebellar nuclei, and in particular the dentate cerebellar nuclei (DCN), were assessed visually by selecting coronal slices from the mean EPI volume where the DCN can be identified as hypo‐intense regions, due to the high iron content that shortens T_2_* (Fig. 1B.a). This was performed for the main effect of movement for each hand at each GF level (see below RFX 1 and 2).

## DETAILS OF THE PERFORMED ANALYSIS ARE GIVEN BELOW

### RFX1: Main Effect of Movement

Main group effects were identified using a one‐sample *t* test on contrast images obtained from the “within‐subject” analysis testing for a main effect of movement. Comparisons of all GFs against baseline were performed for DH or NDH separately.

### RFX2: Main Effect of GF

Main group effects were identified using a one‐sample *t* test based on contrast images from the “within‐subject” analysis at each GF level for DH, NDH, and fNDH. This corresponds to a test for simple main effects of movement under each GF or complexity level and contextualises the analysis of flipped contrast images implicit in the conjunction analyses below.

### RFX3: Linear Response Analyses

Linear analysis of grip‐related responses was performed to identify increased activations at the higher GF, using a one‐sample *t* test on the within subject linear effect contrast images for the DH or NDH.

### RFX4: Specificity, Lateralisation, and Strength of Activations

Three paired *t* tests were performed to assess (a) specificity, (b) lateralisation, and (c) strength of activations when using the DH or the NDH:

- A paired *t* test between the DH and NDH was performed at each GF to test the specificity of regional activations in relation to (right) hand dominance;
- Comparing contrast images with their corresponding flipped data (i.e., running a *t* test of DH with fDH and NDH with fNDH) enabled us to assess the lateralisation of hand‐dependent responses, i.e. highlighting areas that showed an interaction between hand dominance and hemisphere.
- Comparing contrast images from DH with fNDH, it was possible to compare the strength of activation when using the DH or NDH in corresponding CL and IL regions. This also constitutes an interaction with hemisphere; however it can also be considered as a (simple) main effect in terms of the ipsilateral or contralateral relation to the hand used.

### CA: Common Areas of Activations

Conjunction analyses (Supporting Information Fig. 1) were performed to identify common regions of activation at each GF level. Each of the group contrast images for DH, NDH, and fNDH obtained in RFX2 (at each GF level) were thresholded and used as conjoint masks to perform conjunction analyses, testing for common regions in terms of:

- Common right and left hemisphere activations, independently of handedness, identified by a conjunction of DH and NDH effects (Supporting Information Fig. 1a).
- CL and IL hemisphere activations common to both DH and NDH, assessed by a conjunction of fNDH and the DH effects (Supporting Information Fig. 1b).

### Effect Size

In addition, the voxel by voxel (unstandardized) effect size of the group level statistical analyses was calculated and masked with the significant thresholded activations (for the purpose of illustrations—using a voxel level threshold of *P* < 0.001—corrected at the cluster level) to generate significant effect size maps.

## RESULTS

### Task Performance

All subjects performed the task adequately using either hand (Table I, Supporting Information). Also there was no effect of the lateralization scores as measured by the handedness tests on the group findings.

The main results of this study are reported in the first instance as an overview, based on main regional designations, i.e. primary motor, premotor, parietal, visual, and cerebellar areas. Following this, the results of each analyses are described in detail.

### Primary Motor Area (M1)

The primary motor area (M1) CL to each hand was activated at each GF level regardless of the used hand. The linearity analyses revealed, using either hand, that the CL M1 was increasingly involved with increasing GF levels. Using the conjunction analyses, DH CL M1 was mirroring the NDH CL M1 and the right M1 was also commonly shared during both the NDH and DH task.

### Premotor Areas and SMA

**Figure 2:**
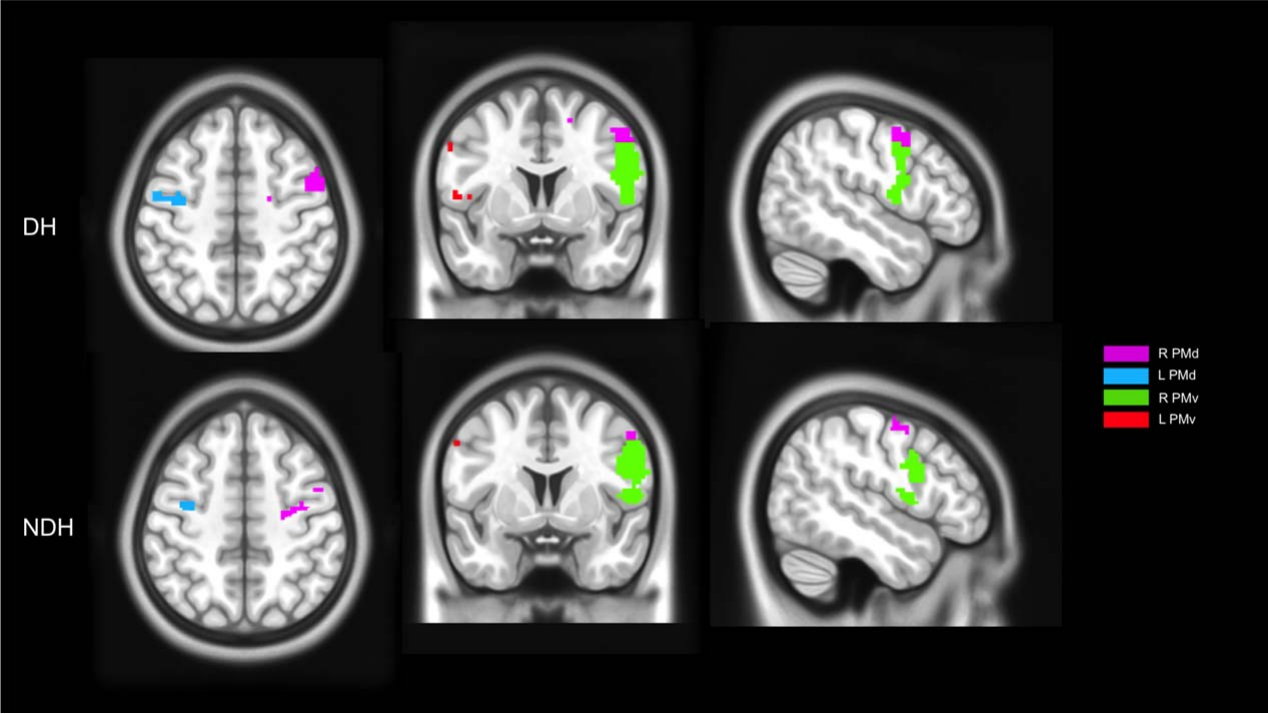
Illustration of activations of main effect of movement within the dorsal (PMd) and ventral (PMv) premotor cortex when using either DH or NDH. These maps were generated by masking significant activations (using a threshold of *P* < 0.001, corrected at the cluster level, for the purpose of illustration) with ROIs localized in the premotor regions using the HMAT template. In the map right is right. [Color figure can be viewed in the online issue, which is available at http://wileyonlinelibrary.com.]

The premotor cortex was more lateralized to the right hemisphere at the highest GF levels using either hand. Using the NDH, as the GF increased, the right PMv and PMd activations increased too, as shown by the linearity analysis. Using the conjunction analyses, the premotor cortex was commonly activated contralaterally to each hand. The conjunction analyses showed that overall the right PMv was commonly activated between the two hands. Figure 2 shows examples of activations in PMv and PMd using the DH and NDH, respectively. Moreover, the CL supplementary motor area (SMA) was activated using the DH at each GF while the IL SMA was activated (significantly) at the highest GF level. Using the NDH, SMA was activated bilaterally especially at GF level of 40%, where there was a high anatomical probability detection of the SMA. There was no specificity or lateralization in SMA using either hand. SMA was commonly shared between activations produced by the two hands, especially at the highest GF level.

### Parietal Cortex

**Figure 3:**
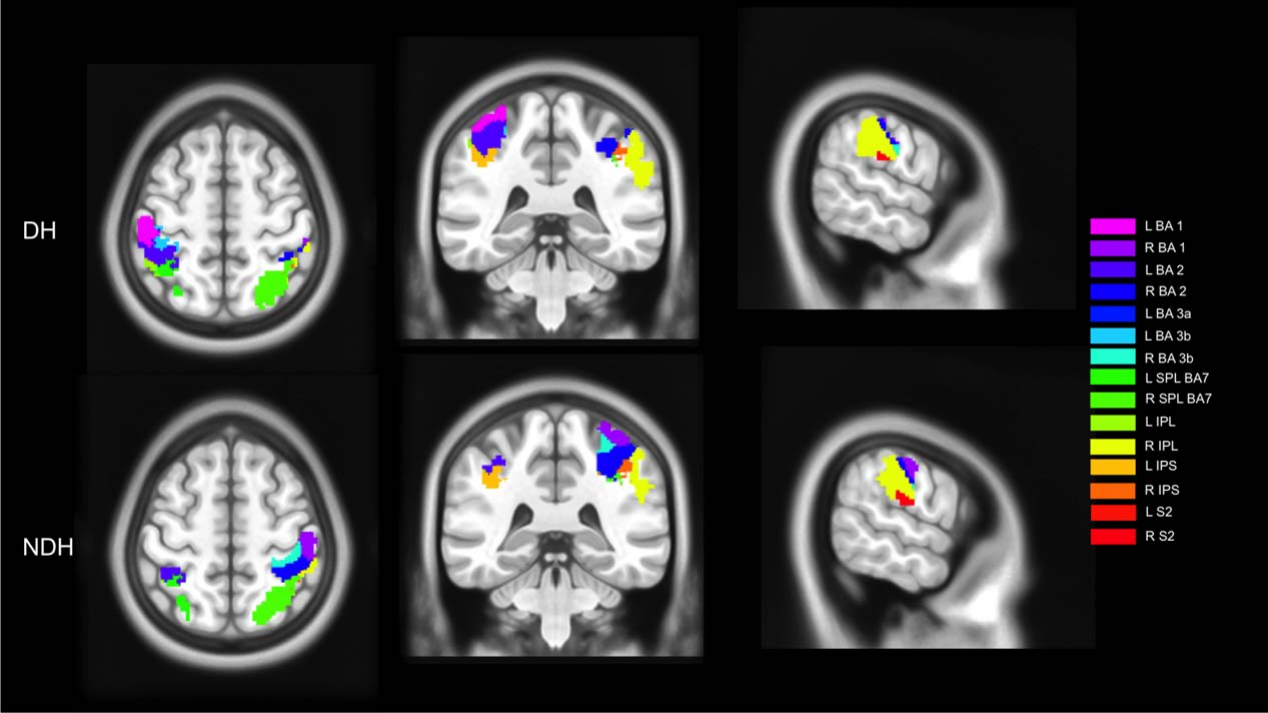
Illustration of activations of main effect of movement within 15 subregions of the parietal lobe when using DH or NDH. These maps were generated by masking significant activations (using a threshold of *P* < 0.001, corrected at the cluster level, for the purpose of illustration) with ROIs localized in the parietal regions. In this convention, right is right. Abbreviations are: BA, Brodmann area; L, left; R, right; SPL, superior parietal lobule; IPL, inferior parietal lobule; IPS, anterior intraparietal sulcus; S2, secondary somatosensory cortex. [Color figure can be viewed in the online issue, which is available at http://wileyonlinelibrary.com.]

The primary somatosensory areas (BA 1, 2, 3) were lateralized to the CL hemisphere to each hand and at each GF levels. The secondary somatosensory cortex (S2) was defined using the cytoarchitectonic software as areas corresponding to the parietal Operculum (OP1‐OP4) (Eickhoff et al., 2006a, 2006b,2007a,^2010^). Our result indicates that during the main effect analyses, the DH activated S2 bilaterally, while it was lateralized to the right hemisphere using the NDH. Qualitative analysis of this region showed that strong bilateral activation using the DH was observed at the highest GF level. In addition, the linearity analyses showed that both hands produced increased activations mainly in BA 3b but also part of BA 2, CL to each hand. Moreover, there were no common regions (hemispheric conjunction analysis) between both hands in the primary somatosensory cortex (i.e., those regions were mainly contralaterally activated). On the other hand, the right S2 was commonly shared at each GF level between the DH and NDH. Looking at higher parietal cortical areas [intraparietal sulcus (IPS), the inferior (IPL) and superior parietal lobule (SPL)] in the right hemisphere, the IPL was commonly activated using both hands. As the GF level increased, the SPL (BA 7A) was commonly detected in the right hemisphere, too. The DH activated bilaterally the IPS, especially at the lowest GF level. Also both hands commonly and constantly activated the IPS in the right hemisphere at each GF level. At the highest GF, the posterior parietal cortex was bilaterally engaged using either hand. Figure 3 shows examples of activations in 15 parietal areas using the DH and NDH, respectively.

### Visual Pathway Areas

There were significant activations in the primary visual and extrastriate visual areas. The extrastriate visual areas (namely V4 and V5) were activated regardless of the different GF levels or the hand used (Supporting Information—Table IX; common areas). In addition, the fusiform gyrus was also mostly involved at each GF level using either hand. In the linearity results, none of the visual areas were detected using either hand.

### The Cerebellum

The main findings were that at each GF, the IL anterior cerebellum and even more the superior posterior cerebellum were strongly activated using either hand. As the GF increased, the IL anterior cerebellum was increasingly recruited (using the linearity analysis), especially when using the NDH. Both hands activated the CL cerebellum with patterns dependent on the GF level. For example, the DH activated the CL cerebellum at the middle GF level only, while the NDH produced continuous bilateral activations at each GF level. This CL activation was mainly localized in the posterior lobe. Figure 1A shows activated cerebellar network overlaid on the SUIT flattened template. In addition, we identified activations in the deep cerebellar nuclei. As shown in Figure 1B.b–d, the DCN were increasingly activated, mainly ipsilaterally to each hand, as the GF level increased from 20 to 60% of MVC. The predominance of ipsilateral activations was also confirmed by the main effect of movements (Fig. 1B.e). It was also clear that the NDH activated part of the contralateral cerebellar nuclei at the highest GF level (Fig. 1B.d, blue area).

A descriptive of the results for all test for main effects and interactions is provided below. Further details can be found in the Supporting Information in the form of tables, organised into motor areas, nonmotor areas, and cerebellum.

### RFX1: Average Main Effect of Movement

The results show that both hands activated well known visuomotor areas, such as the pre/post central gyri (primary motor (M1) and somatosensory (S1) cortices), S2, SMA, the PMd and PMv, IPL, SPL, and the cerebellum. The NDH was associated with larger clusters located in the primary motor cortex and the cerebellum, relative to the DH. Looking specifically at premotor regions, PMd was activated largely contralaterally to each hand, while the right PMv was largely activated independently of the hand used (Fig. 2).

### RFX2: Main Effect of GF

**Figure 4:**
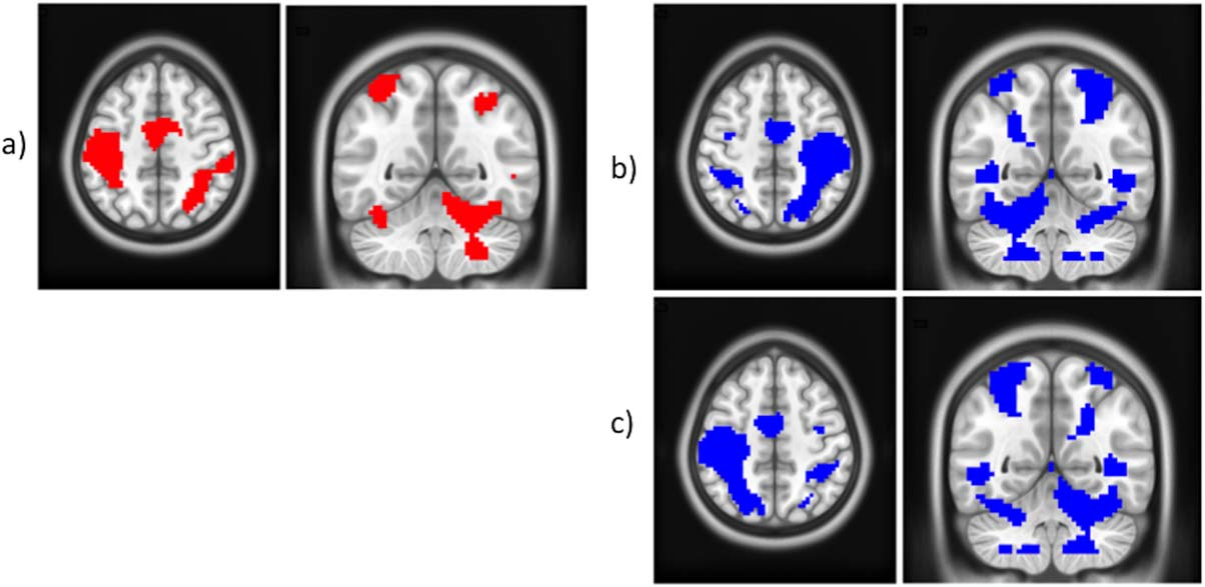
One sample *t* tests at the group level and 60% GF level for (**a**) DH (red); (**b**) NDH (blue); (**c**) fDNH (blue). All clusters are corrected for multiple comparisons after using a threshold of 0.001 (uncorrected) at the voxel level. In the images, right is right and left is left; axial cut at *z* = 50; coronal cut at *y* = −54. [Color figure can be viewed in the online issue, which is available at http://wileyonlinelibrary.com.]

Figure 4a–c show example results using the group one‐sample *t* test for DH, NDH, and fNDH, respectively, at the highest GF level (60% of the subject's MVC).

The main effect analysed for each individual GF level confirmed the engagement of primary motor, visual, and cerebellar areas. A qualitative comparison of the results indicates that these activations increase with GF (quantitative comparisons and linearity are reported in RFX 3–4 and CA). For example, at the highest GF level greater activation is seen in fronto‐occipital areas, with both the IL and CL cerebellum contributing as the GF increases in particular when using the NDH. These simple main effects indicate an interaction between GF and using the NDH in producing differential responses, particularly at high force levels. This is an example of how the complexity of the task (GF) contextualises—or is contextualised by—the complexity of execution (DH versus NDH).

### RFX3: Linear Response Analyses

**Figure 5:**
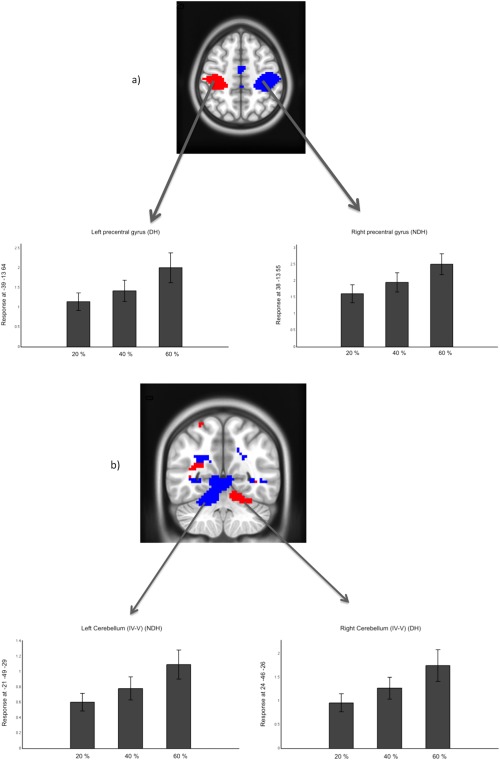
One sample *t* tests at the group level of Linear effect responses using DH (red) and NDH (blue) in (**a**) precentral gyri; (**b**) cerebellum IV–V. All clusters are corrected for multiple comparisons after using a threshold of 0.001 (uncorrected) at the voxel level. In the images, right is right and left is left; axial cut at *z* = 50; coronal cut at *y* = −54. [Color figure can be viewed in the online issue, which is available at http://wileyonlinelibrary.com.]

The linear response group analysis statistically compared activations at 20 and 60% GF. The results of this quantitative comparison indicated an increased response in the CL pre/post central gyri [primary motor (M1) and somatosensory (S1) cortices] using either hand. This included the anterior and posterior parts of BA 4 (4a, 4p, respectively) and BA 3b. Performing the task with the NDH was associated with activations proportional to GF localised in the IL cerebellum (lobules I–IV, V, VI) and in other CL deep grey matter areas (e.g., posterior cingulum, thalamus, hippocampus), premotor cortex (BA 6), as well as the Vermis of the cerebellum. As opposed to the DH, the NDH showed that the right PMd and PMv were linearly increasing, as detected quantitatively using the linearity analysis, with increasing GF levels. These additional activations reflect an interaction between (linear) increases in GF and the use of the DH versus NDH. An example of the linearity result is illustrated in Figure 5.

### RFX4: Specificity, Lateralisation, and Strength of Activations

Overall, specificity of activations was localized in the CL pre/post central gyri and in the IL cerebellum, while lateralisation of activations was more confined to right hemisphere regions (e.g., insula and inferior frontal gyrus). In detail:

#### Specificity

**Figure 6:**
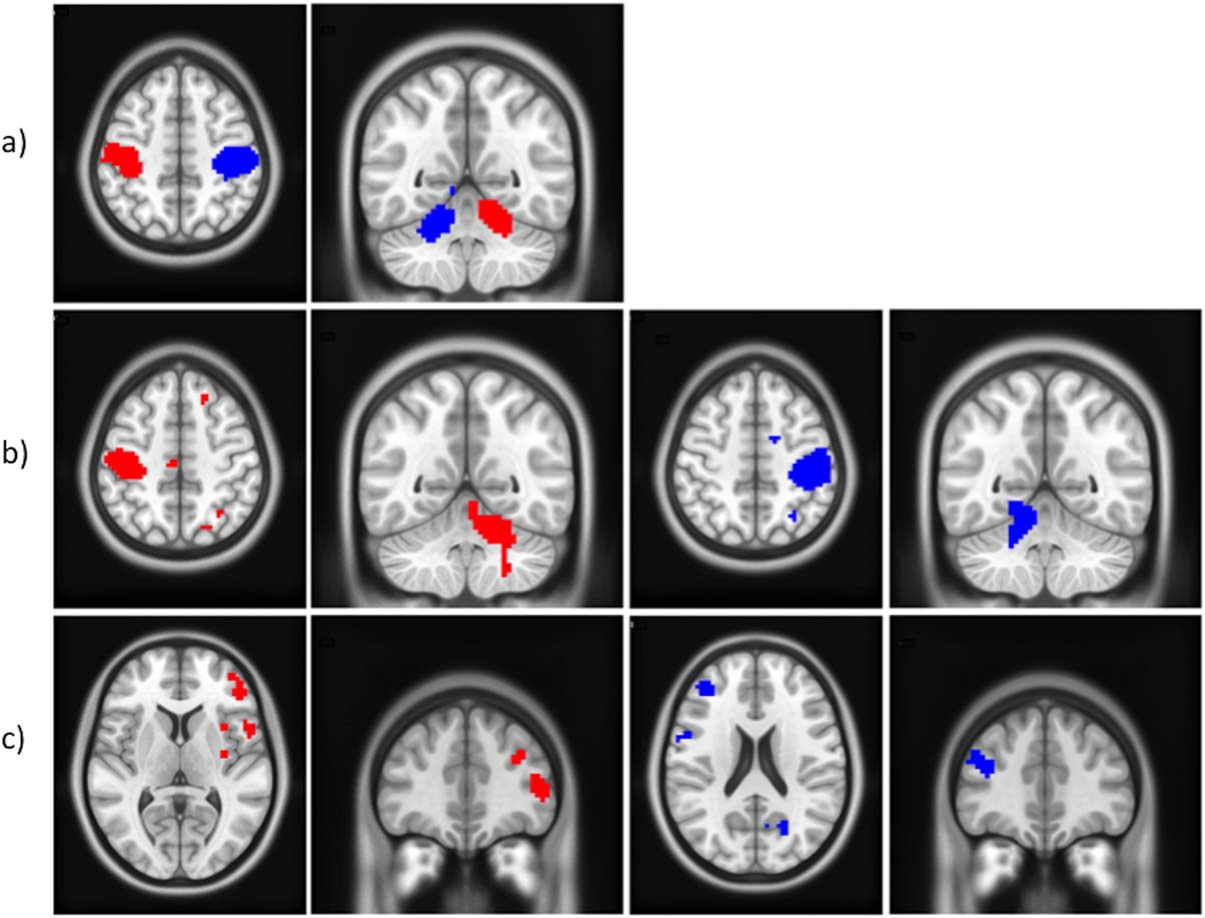
Paired *t* test analyses at the group level and 60% GF level to compare the DH (red) with NDH (blue), or fNDH (blue). (**a**) Specificity of regions (by comparing DH and NDH); (**b**) Lateralisation of regions by comparing DH or NDH versus their flipped images; c) Strength of activations by comparing DH and fNDH. All clusters are corrected for multiple comparisons after using a threshold of 0.001 (uncorrected) at the voxel level. In the images, right is right and left is left. [Color figure can be viewed in the online issue, which is available at http://wileyonlinelibrary.com.]

With this analysis, differences between activations obtained with DH and NDH tasks were tested at each GF level (Fig. 6a) to establish which areas are specific to using the DH or NDH. At each GF level hands specific responses were seen mostly in CL M1 (BA 4a for the DH; 4p for the NDH), CL S1 (BA 3b, 1). The IL cerebellum (lobule VI) proved to be highly specific (i.e., always present independently of GF) for the DH while BA 6 for the NDH, respectively.

#### Lateralization

To assess whether certain areas were specific to either CL or IL regions, paired *t* tests compared responses for each hand with their corresponding flipped images were performed. In this analysis, if a region activated bilaterally, it would not survive the comparison. For the DH and for all GF levels (Fig. 6b), the most consistently activated regions were the CL M1 (BA 4a) and the IL cerebellum (lobule VI). The CL premotor region (BA 6) and S1 (BA 1) were also lateralized (although the cytoarchitectonic probability of these regions was lower at the middle and highest GF, respectively). The IL SPL (BA 7) was lateralized only at GF of 20% while the IL inferior frontal operculum (BA 44) was lateralized at GF of 40 and 60%. For the NDH and at each GF level (Fig. 6b), consistent lateralized regions were the CL S1 (BA 1), SMG, and Rolandic operculum (OP 1, 2) (S2). Lobule VI of the IL cerebellum was lateralized only at the lowest and middle GF levels. Additionally, the CL putamen and insula were activated at the lowest and highest GF levels, respectively. The lateralization analysis confirmed that IPS and S2 were more lateralized to the right hemisphere when using either hand.

#### Strength

Comparing DH and fNDH activations (Fig. 6c) showed that there was a noticeable difference in strength of activation (greater for DH) in areas including the right inferior frontal operculum and triangular as well as the SMG; this was especially true when using the DH at the lowest GF. At the highest GF level, the DH showed also increased activation in the right precentral gyri, while greater activation for the fNDH was only noticeable at the middle GF level in the left middle frontal gyrus.

#### CA: conjunction analyses

##### Conjunction analysis (a)

With this analysis it was possible to show the common regions of responses, in both hemispheres, between the DH and NDH at GF levels of 20, 40, and 60%, respectively.

**Figure 7:**
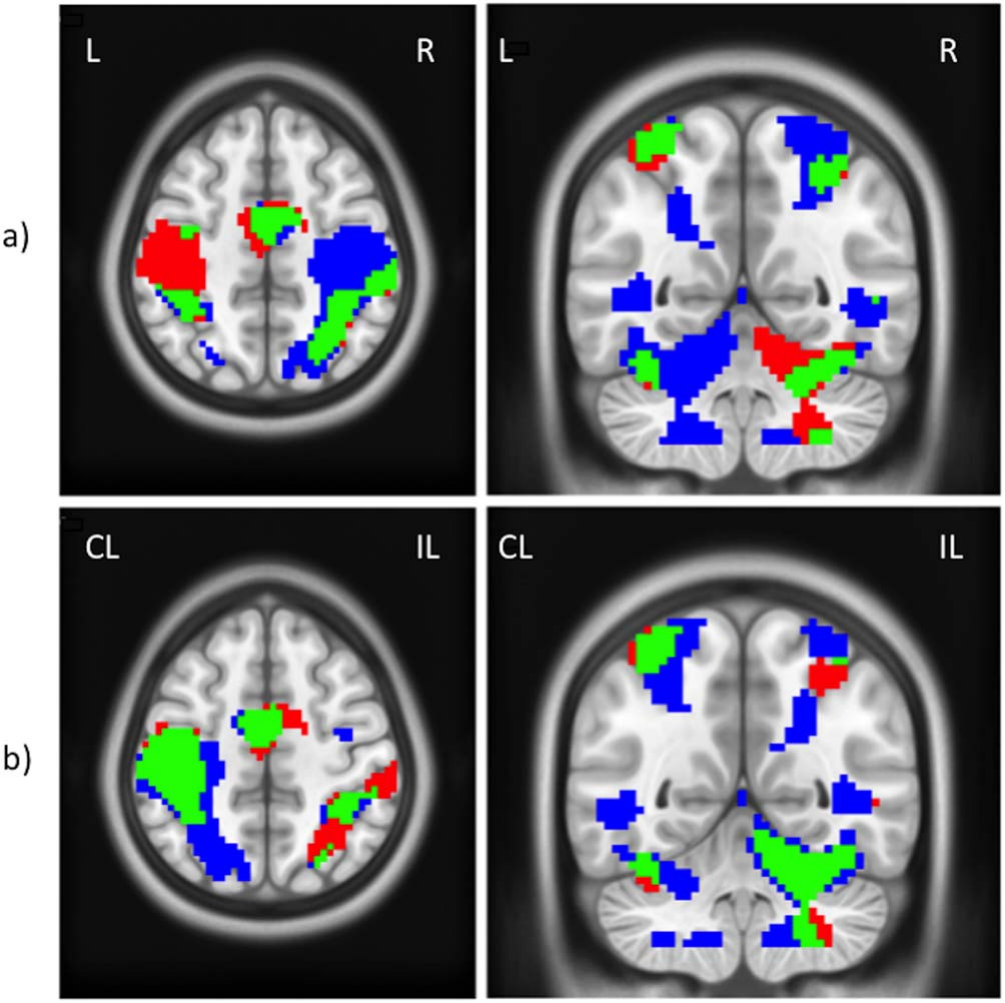
Conjunction analysis—with common areas illustrated in green—at the group level and the 60% GF level of (**a**) DH (red) and NDH (blue); (**b**) DH (red) and fNDH (blue). All clusters are corrected for multiple comparisons after using a threshold of 0.001 (uncorrected) at the voxel level. Axial cut at *z* = 50; coronal cut at *y* = −54. [Color figure can be viewed in the online issue, which is available at http://wileyonlinelibrary.com.]

Responses common to both hands and at each GF level were seen in the right hemisphere and included: SMA—BA 6, S2, SMG, IPL, BA 44, lobule VIIb of the cerebellum, visual areas in the occipital and temporal regions (e.g., V4; V5) and the fusiform gyrus. Figure 7a is an example of this analysis performed at a GF of 60%. On the other hand, the common areas seen at each GF level in the left hemisphere were localised to the visual cortex (V5)—located in the inferior/middle occipital and temporal gyri. In addition, using the premotor‐specific template, the right PMv was commonly activated regardless of the used hand.

In addition, there were activations specific to two GF levels. For example, regions commonly activated in the right hemisphere at GF levels of 20 and 40% included SPL (BA 7A) and the anterior intraparietal sulcus (IPS) (hIP3), while regions commonly activated at GF levels of 40 and 60% included left SMA (BA 6), PMd, right cerebellum (lobule VI), and right parietal operculum (OP 4).

Furthermore, there were regions that were found to be common at specific GF levels: at a GF of 20% the right anterior IPS (hIP2); at a GF of 40% lobule VIIIa in the right cerebellum and lobule IV in the left cerebellum; and at a GF of 60% the left IPL, SPL, and the middle cingulate cortex.

See the Supporting Information Tables VIIIa–c for further details.

##### Conjunction analysis (b)

Supporting Information Tables IXa–c give the areas of activation in the CL and IL hemispheres common to motor tasks performed with either hand at GF levels of 20, 40, and 60%, respectively. Figure 7b demonstrates an example of this analysis at a GF level of 60%.

Considering all the GF levels, common CL regions were detected in S1 (BA 1, 3b), IPL, SMA, the premotor cortex (BA 6) (PMd), inferior temporal and occipital gyri (V5), while the IL regions included the middle, temporal, and inferior occipital gyri (V5, V4). Common IL activations for the lowest (20%) and middle (40%) GF levels were only detected in lobule VIIb of the cerebellum, while for the middle (40%) and the highest (60%) GF levels the IL cerebellum (lobule VI) was observed. No CL regions were exclusively detected at the lowest (20%) and middle (40%) GF levels. On the other hand, there were specific CL regions common to the middle and highest GF levels including the SMG and M1 (BA 4a).

Furthermore, there were regions that were commonly shared between the DH and fNDH at one of the three GF levels, including the CL IPS and BA 4p at GF of 20%, and lobule VI of the CL cerebellum at GF of 40%. At GF of 60%, lobule VIIIa of the IL cerebellum, the CL Rolandic operculum (OP 3), and the CL SPL were common to the DH and fNDH.

The findings of the conjunction analyses (a) and (b) show that the NDH task activates bilateral regions such as the SMA and cerebellum (VI), which are common to areas activated by the DH at the highest and middle forces, respectively.

In addition, as already described above and summarized in Figure 1B, we identified mainly IL activations in the DCN, although at the highest GF the NDH activated part of the CL DCN too.

### Effect Size

**Figure 8:**
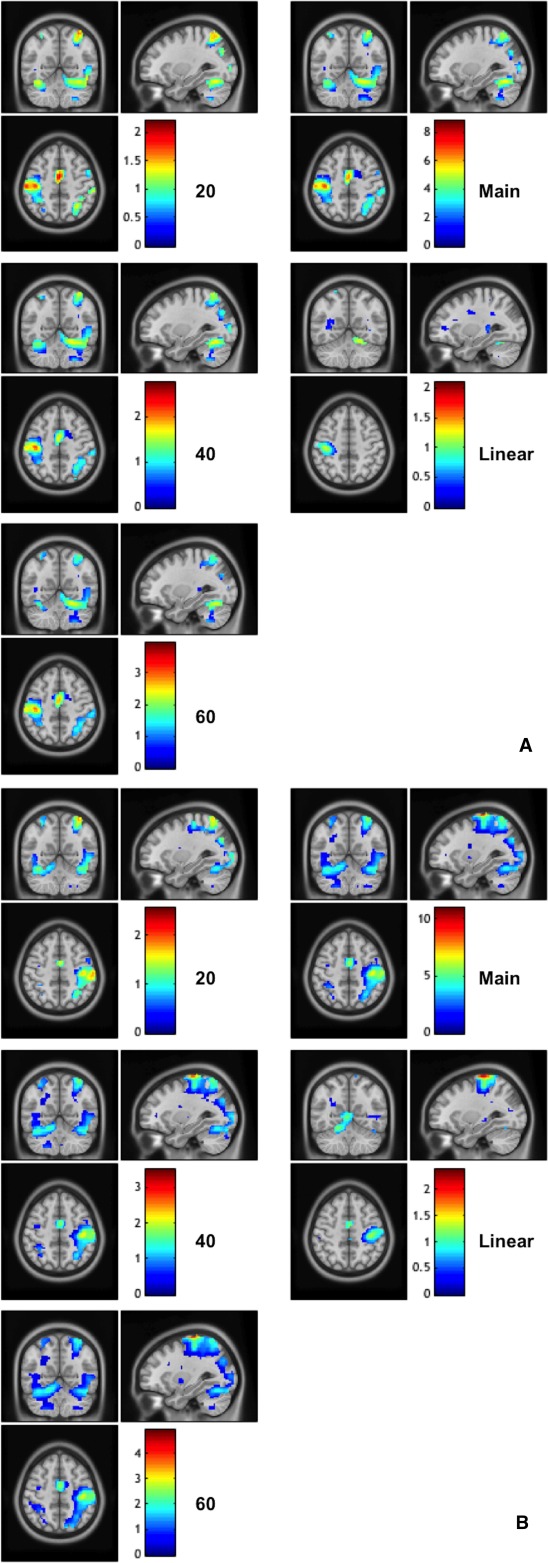
The calculated (unstandardized) effect size maps when using the DH (frame A) and NDH (frame B). The effect sizes are reported for each individual analyses at each force level (20, 40, and 60%), the main effect of forces and the linear activations. In the map right is right. [Color figure can be viewed in the online issue, which is available at http://wileyonlinelibrary.com.]

The calculated effect size values are reported voxel by voxel on Figure 8, showing generally that highest effect size was seen during the main effect of movement and at the same time that the effect size increased as the force level increased. Furthermore, the calculated effect size (or contrast estimates) for two specific regions of interests, i.e., the primary motor area and the anterior cerebellum, when using both DH and NDH, are shown together with examples of linearity response to GF in Figure 5.

## DISCUSSION

This study characterised the effects of performing motor tasks of different degrees of complexity and the interaction with its executive complexity in right‐handed healthy volunteers. The paradigm, performed with either the DH or NDH also required a high degree of visual attention and processing. The overall message is that the complexity of the paradigm (applying increasing GF levels) interacts with the complexity of the execution of the task (DH versus NDH), with differential and increased enrolment of extensive motor and nonmotor areas, the right hemisphere and the cerebellum.

Dynamic power grip designs, similar to the one used in this study, have previously been shown to be a powerful way to investigate motor functions in healthy controls, aging, and stroke disease [Boudrias et al., 2012; Talelli et al., 2008; Ward et al., 2006]. In the current study, task performance was consistent using either hand for all subjects, both in terms of reaching the required GF target and maintaining the grip force for the specified duration. Data was put through a statistical analysis plan that was innovative in its conception: the cascade of the analysis tests was specific to the study questions, starting from generating fixed effect analyses (within subjects) with GLM, then designing a series of RFXs to elucidate and disentangle specific interactions between task and performance complexity, and finally using CA for determining common regions of activation.

### Main Effect of Task

The main effect of using the DH, irrespective of GF level, was generally in line with previous studies of the power grip dynamic motor task [Ehrsson et al., 2000; Keisker et al., 2009; King et al., 2014; Kuhtz‐Buschbeck et al., 2008; Ward and Frackowiak, 2003]. This study also demonstrated that using the NDH recruits larger activations as compared to the DH, mainly in motor and visual areas in fronto‐parieto‐occipital regions, as well as in the cerebellum.

### Complexity of the Task and its Execution

The complexity of neuronal processing in this study combined two factors involved in every‐day functions: the task complexity induced by the level of the applied force (from 20 to 60% MVC) and the executive complexity induced by the use of dominant or nondominant executive systems (DH or NDH). Previous fMRI GF studies focused on the effect of different forces applied using the DH [Keisker et al., 2009; Kuhtz‐Buschbeck et al., 2008; Spraker et al., 2012; Talelli et al., 2008; Ward et al., 2006, 2007, 2008]. In these studies, the regions responded in a linear fashion and were mainly localised with the CL M1 and IL cerebellum. For the DH hand, we showed a linear increase in activation with GF in the CL subdivision of M1 or BA 4 (BA 4a and 4p), and S1 (BA 2, 3b), while the effect in the cerebellum could be observed at a lower statistical threshold (*P* < 0.001) (Fig. 5b). The cytoarchitectural subdivision of BA 4 into BA 4a and 4p has been linked to the fact that these subareas are also functionally distinguished [Geyer et al., 1996]. Recently, we also demonstrated that BA 4p but not 4a responds to increasing GF levels with a BOLD signal changing in a complex nonlinear fashion [Alahmadi et al., 2015]. In line with this finding, previous studies have suggested that different neuronal populations could behave differently within a cortical area [Alahmadi et al., 2015; Ashe, 1997; Ward and Frackowiak, 2003]. Moreover, it has been shown that BA 4p but not BA 4a is highly modulated by attention [Binkofski et al., 2002]. Although this sort of differentiation was not the focus of the current study, it is possible that attention‐modulated subject's response might explain a differential activation of BA4a from BA4p. This specific result can be considered as reflecting a task‐dependent behaviour rather than execution modulated behaviour, in line with the hypothesis that attention to performance of the task—i.e., reaching the requested GF—is similar for both hands. Moreover, previous studies have shown that the IPS is also involved in modulating attention [Coull and Frith, 1998; Majerus et al., 2007; Silk et al., 2010], indicating that there is a possible functional connection between these two regions, especially when finely varying GF levels. This could be an interesting topic for future investigations.

During the NDH performance and using the linearity analysis, contrasting the DH network, several areas demonstrated greater or additional activations outside the CL M1 and IL cerebellum; these included the CL premotor cortex (PMv and PMd), hippocampus, and thalamus, which are thought to play a role in higher‐order motor control processes. For example, the premotor cortex (BA 6) has been shown to be involved in executing and planning movements, as well as in motor control of tasks requiring spatial attention [Begliomini et al., 2008; Simon et al., 2002; Van der Lubbe and Abrahamse, 2011]. The findings of the present study suggest that during NDH tasks, the increased GF is linked with a stronger activation not only of areas involved with movement control, but also in more cognitive structures such as the hippocampus and prefrontal areas, which are known to respond to increased attentional demands [Ashe, 1997; Keisker et al., 2009; Ward and Frackowiak, 2003]. In fact, the prefrontal cortex has been reported as the area responsible for mediating premotor cortex activities [Rowe et al., 2002].

### The Role of Each Hemisphere

One question this study is contributing to answer is whether activations are hemisphere specific, bilateral or depend on the hand used for the movement. Understanding the specificity and lateralization of the activation patterns can help understanding the role of different brain regions in the proposed motor task. Looking at both cerebral hemispheres—and at each GF level—with conjunction analyses this study showed that the right hemisphere housed regions activated at all GF levels such as the precentral gyrus, SMG, fusiform, and visual areas (e.g., V4). Most of these regions are nonprimary motor areas that are involved in attentional processing and sensory‐spatial integration. It has been shown that the right hemisphere is crucial for visual target detection as well as for visual spatial attention in which frontal, parietal, and temporal areas play a key role [Neely et al., 2013; Shulman et al., 2010; Woolley et al., 2010]. This is in line with the engagement (seen in our data) of the posterior parietal cortices, namely, the right SPL and the IPL, which have been shown to play a role in the integration of visual information—thus allowing online motor control through modulation of prefrontal motor areas [Hamzei et al., 2002; Marconi et al., 2001; Neely et al., 2013]. The predominance of right hemisphere regions is also supported by evidence that the right hemisphere is specifically involved in grasping networks [Begliomini et al., 2008] and by studies showing that performing sequential movements with the NDH produces greater activations in the CL hemisphere (hence here the right hemisphere) as compared to the DH [Jäncke et al., 1998; Ng et al., 2008; Seong‐Gi et al., 1993]. It has been argued that the recruitment of the motor cortex of right‐handers increases when using their NDH and that the more skilled and more widely used cortex requires less effort and, hence, less recruitment and signal [Amunts et al., 1996; Jäncke et al., 1998; Ng et al., 2008]. Furthermore, in this study we looked at the network that is shared between the DH and NDH. This is distinct from looking at an individual pathway. Based on these results, it is arguable that the right hemisphere is more engaged in the current cohort of right‐handed subjects regardless of the hand used when a dynamic power grip movement is visually controlled to produce different GF levels.

On the other hand, the left hemisphere did not contain a single (significant) region that was commonly activated at every GF. Compared to previous studies that claimed the prominence of the left hemisphere [Grafton et al., 2002; Marian, 1973; Seong‐Gi et al., 1993], this work interrogated the task complexity together with its execution using the DH/NDH, therefore the results are reflecting the behaviour of a shared network between the DH and NDH and not simply the linearity of the functional response with increased GF. Compared to finger tapping [Reddy et al., 2000; Cramer et al, 1999; Verstynen et al., 2005], our task required whole hand movements squeezing a ball that invokes somatosensory feedback, in addition to attending to external visual cues, i.e. engaging an additional ecologically‐relevant cognitive component [see Kuhtz‐Buschbeck et al., 2008; Noble et al., 2013, for more discussion]. When analysing the effects of applying increasingly greater GF the results suggest an increase in grey matter recruitment, especially in the left hemisphere. It has previously been shown that the left hemisphere is dominant for motor planning [Janssen et al., 2009] both for right and left‐handed subjects [Janssen et al., 2011]. Here, it was found that secondary sensorimotor areas, such as the left premotor areas, were significantly more active at the highest GF levels than at the lowest GF. Previous studies suggest that one of the key roles of the premotor cortices is in movement planning [Grafton et al., 2002; Seong‐Gi et al., 1993; Verstynen et al., 2005]. This study supports these findings and also suggests a key modulatory role for task complexity (as opposed to execution complexity) on left hemisphere premotor cortex activity.

### Regional Involvement

Amongst the various regions highlighted by the analysis, it is worth discussing the activation of the right hemisphere primary motor (M1) area, parietal and premotor regions, visual areas and the cerebellum, as examples of the complexity of the motor network. The readers are referred to the tables in the Supporting Information for further details of the complexity of the network involved in this task, both with increased GF and with increased execution difficulty. Future studies should investigate functional and structural connectivity of the grasping network, learning from the current results to establish hypothesis‐driven models and to examine how motor, visual and associative cortical and cerebellar areas may interplay especially at high GF, both for DH and NDH tasks.

#### Primary motor

Amongst the primary motor areas, it is worth discussing the right M1 because it was activated not only when using the NDH (consistent with an expected CL activation of the primary motor cortex), but also during the DH task. This result confirms the belief that there is a role for the IL M1 in performing unimanual DH tasks in right‐handed subjects, which has previously been reported using various techniques, including transcranial magnetic stimulation (TMS) [Bawa et al., 2004], intracortical microstimulation (ICMS) [Brus‐Ramer et al., 2009], and near‐infrared spectroscopy (NIRS) [Shibuya et al., 2014], as well as fMRI [Hammond, 2002]; however, its function remains poorly understood. It is possible that the right hemisphere M1 is involved in interhemispheric interactions for controlling the applied force [as also shown by Shibuya et al., 2014], but also in processing the visual feedback cue and translating it into movement, consistent with the dominant role of the right hemisphere in visually guided movement. In the study presented here, the right M1, IL to the DH, was highlighted by the conjunction analyses as the exact area that responded CL to the NDH, supporting the hypothesis that the right M1 is communicating with the CL M1 in DH motor tasks, performing the transformation of a visual instruction into a motor action.

#### Parietal and premotor regions

Among the detected regions, a complex activation pattern was observed in the parietal cortex. The primary somatosensory cortex not only showed a main effect of movement but it also showed an extended linear response, especially in BA 3b, between GF and BOLD signal. The activation in this somatosensory region is in line with previous fMRI visuomotor grip studies [e.g., Alahmadi et al., 2015; King et al., 2014; Kuhtz‐Buschbeck et al., 2008]. What is more interesting is that, compared to the other grip types, the dynamic power grip generates activations that are uniquely localized in BA 3b, as shown by using an activation likelihood estimation analyses [King et al., 2014]. In our study, and regardless of the hand used, area BA 3b responded in a uniquely linear fashion, suggesting that this area may play a key role in mediating GF level‐dependent processing. This behaviour is very similar to that of M1 and is in line with published evidence of a modulatory effect of the primary sensory cortex on primary motor areas [Petrof et al., 2015], suggesting a direct connection between these two areas, with afferents from S1 (here 3b) to M1. This seems particularly relevant for performing the task at higher GF levels [Keisker et al., 2009], but not for the complexity of the task.

One of the important findings is the commonly shared activation between the two hands in the right IPS, and IPL (see “the role of each hemisphere” section). These regions work as a bridge between the visual cortex, especially the extrastriate regions, highly involved in our study, and the premotor area, which then communicates with the primary sensory and motor areas [Hamzei et al., 2002; Keisker et al., 2009]. Supporting this statement, previous studies showed that these regions are highly involved in controlling on‐going visually guided movements [Alahmadi et al., 2015; Ehrsson et al., 2001; Frey et al., 2005; Keisker et al., 2009; Martin et al., 2011; Vaillancourt et al., 2003]. Our study suggests that the right PMv and the posterior parietal cortex are engaging in the same way regardless of the used hand. This can be seen using the conjunction analyses and supports the involvement of the premotor parietal circuitry in the visually‐guided grasping network. Moreover, the involvement of PMv as compared to PMd using either hand is in line with previous studies suggesting that the PMv is mainly engaged in gripping and grasping movements [Grefkes and Fink, 2005; Rottschy et al., 2012]. Similarly, the involvement of the anterior IPS (as a shared region using either hand and at each GF level) reflects its role in visually guided grasping movement, confirming previous literature [Frey et al., 2005; Grefkes and Fink, 2005; Hamilton and Grafton, 2006; Rice et al., 2006; Tunik et al., 2007]. It is also interesting to see that most of the parietal areas get activated bilaterally at the highest GF level. From a task point of view, this suggests that the left hemisphere higher order (associative) parietal areas have a modulatory role for task complexity, similarly to the premotor region.

### Visual Pathway Areas

The use of visual cues to guide movement has been shown to activate a number of regions involved in visual processing, such as the IPL, SPL, fusiform, middle occipital, and temporal gyri [Kuhtz‐Buschbeck et al., 2008; Noble et al., 2013], and this pattern of activation has also been observed in our study. There were constant activations detected in higher order visual areas (the extrastriate visual areas, V4 and V5) as well as in the fusiform gyrus. These activations are known to be related to the transformations and control of guided cue along the dorsal stream pathway to the posterior parietal cortex [Driver and Mattingley, 1998; Lloyd et al., 2006]. The constant activations of areas V4 and V5 could be related to modulation of attention (V4) [Bressler and Silver, 2010] or the guidance of movement during grasping (V5) [Born and Bradley, 2005]. It is also interesting to note the common activations between DH and NDH in the fusiform gyrus, which is coherent with the knowledge of a link between this gyrus and the visual pathway along the ventral pathway, involved especially during colour processing [Zeki and Marini, 1998]. This is in line with the colorful nature of the visual stimuls used in the study and suggests that not only the presence of a visual stimulus but also its nature modulates the pattern of the activation of the visual system during visually guided motor tasks. Conversely, visual areas activations were not impacted on by task performance or execution performance, but were constant in all conditions of the study. These observations suggest that during visually guided motor tasks, visual areas are not modulated by motor task complexity but only by the characteristics of the visual cue.

### Cerebellum and Deep Cerebellar Nuclei

The other interesting result of this study is the increasing involvement of the posterior and right cerebellum with an increased complexity of the task and its execution. The involvement of the IL motor cerebellum is expected as a result of the main effect of movement. In this study activations in lobules VI, VIIb, and VIIIa were also observed, in agreement with previous sensorimotor studies where the posterior cerebellum was shown to play an important role in coordinating fine motor movement and performing executive functions [Stoodley and Schmahmann, 2009], both needed to correctly perform the motor task used in this study. The finding that the right cerebellum is activated at each GF level and for both DH and NDH points to its key role in motor planning, as well to the presence of a lateralized functional specialization of the cerebellum. The finding of a bilateral involvement of the cerebellum (especially lobule VI) is interesting and supported by the two conjunction analyses and is also in line with a recent report [Holmström et al., 2011], which interpreted this result as an engagement of the cerebellum for error tracking [Imamizu et al., 2000]. Indeed, the feedback visual signal used in the present study and the need for performing the squeezing movement at specific GF levels involved tracking errors and supports the involvement of the bilateral cerebellar activity regardless of the hand used. These results warrant future studies to test specific cerebellar functions, such as timing and sensory prediction, involving higher cognitive processing [D'Angelo and Casali, 2013; D'Angelo et al., 2013].

In addition, we investigated the activations of the deep nuclei of the cerebellum. Despite their importance, activations of these nuclei are very rarely mentioned in fMRI motor studies probably because of their small size and therefore the difficulty of visualising them [Diedrichsen et al., 2011; Habas, 2010]. A few groups, though, have focused their attention on the largest of these nuclei, the right and left deep cerebellar nuclei or DCN [Dimitrova et al., 2006; Gao et al., 1996; Habas, 2010]. The DCNs are a convergence point for axons of Purkinje cells coming from large areas of the cerebellar cortex [Diedrichsen et al., 2011] and it is known that they emit fibers that form the bulk of the superior cerebellar peduncles (SCP). It has been shown that the majority of the connections from the SCP are toward associative/non‐motor areas [Palesi et al., 2014]. Therefore, it is not surprising to see that the DCNs are increasingly involved by an increased complexity of the task as well as of its execution, requiring a greater associative and cognitive processing, mimicking the pattern observed also for associative parietal areas. Our finding is in agreement with a previous report [Gao et al., 1996] that showed a greater DCN activation when increasing the cognitive demand of the motor task, requiring processing of sensory feedback as well as motor functions. It is also interesting to see that, when using the NDH, there is an increasing activation of the contralateral nucleus, supporting a similar pattern of findings as in other cerebellar and cortical areas and possibly suggesting a functional specialization of the DCN nucleus ipsilateral to the DH for motor control.

### Methodological Considerations and Limitations

Our cohort comprised only right‐handed subjects. Future studies are needed to assess findings in left‐handed subjects, where an extra layer of complexity will be added to the network. Furthermore, the results of this study apply to a relatively young group of volunteers, hence it would be interesting to study cohorts with larger age range, as brain activation is associated with age [Talelli et al., 2008; Ward and Frackowiak 2003]. The effect sizes reported in this study are consistent with an event related design of a number of subjects typical of most visuomotor fMRI studies, ranging from 5 to 14 [Ehrsson et al., 2000; Galléa et al., 2005, 2008; Goswami et al., 2011; Keisker et al., 2009, 2010; Kuhtz‐Buschbeck et al., 2008; Spraker et al., 2007, 2012; Sulzer et al., 2011; Vaillancourt et al., 2003]. We refer the reader to [Friston, 2012], for a review of this issue. Also the statistical tests using random effect classical inference analyses (controlling for false positives) conducted in this study are independent (orthogonal).

In terms of the paradigm, an external visually guided cue has been widely used in previous published work [Alahmadi et al., 2015; Galléa et al., 2008; Hilty et al., 2011; Keisker et al., 2009, 2010; Kuhtz‐Buschbeck et al., 2008; Neely et al., 2013; Spraker et al., 2007, 2009, 2012; Sulzer et al. 2011; Vaillancourt et al., 2003; Ward, 2004; Ward and Frackowiak, 2003, Ward et al., 2008; Wong et al., 2007]. To underline that such motor paradigms are visually guided, they are often referred to as “visuomotor.”

We used the same colour‐coded feedback signal cue across trials and when using either hand; thus, this feedback is unlikely to confound the complexity of the task as also shown by the relative homogeneity of visual areas activations during the different experimental conditions. Our task requires visual processing and the use of a squeezeball, which involves a non‐negligible degree of tactile sensory processing. Using motor devices similar to that used in the present work is well established in GF studies [e.g., Alahmadi et al., 2015; Keisker et al., 2009, 2010; Kuhtz‐Buschbeck et al., 2008; Sterr et al., 2009; Ward, 2004; Ward and Frackowiak, 2003; Ward et al., 2008]. However, as the principal focus of the current study was to distinguish the effect of GF level on brain networks and the interaction with the complexity of execution with the NDH, it is not possible to directly assess the contribution of different sensory inputs to our results. Detailed maps of the pattern of brain activations due to unimodal tactile and visual exploration of the same objects are available [Man et al., 2015]. Comparing our activations with these maps, it is possible to hypothesise that the postcentral gyrus is mainly activated by tactile stimuli, while the activations observed in the medial and lateral occipital cortex and in the fusiform gyrus are due to the visual stimuli. Areas thought to play a role in multimodal integration, such as the SPL, were also detected in our study, suggesting the need for the integration of visual and tactile information to correctly perform the task.

The range and selection of grip force levels should also be considered. In this study, a range of forces from 20 to 60% of each subjects' MVC was used. This range was chosen for several reasons. Most of the daily common functions require forces of this range [Marshall and Armstrong, 2004]. Moreover, higher force levels could possibly induce fatigability and therefore may not be performed adequately. Also, it is important to stress that this study investigated the visuomotor network in the context of a power grip dynamic task, where all fingers participated in performing the task (as opposed to a precision task where two fingers perform the task or to a finger tapping task). This is important as it has been shown that there are differences and specific activations between the two tasks [e.g., Khorrami et al., 2011; King et al., 2014].

Overall, the current study offers a substantial characterisation of visuomotor processing engaged by a dynamic power grip task executed with the DH and NDH and, at the same time, illustrates a clear differentiation between qualitative and quantitative approaches to fMRI analysis. This study shows that event‐related designs can be powerful (e.g., allowing activations to be detected in the deep cerebellar nuclei) when carefully optimized. This sort of studies is essential as a prelude to hypothesis‐driven connectivity analyses that characterise task/execution complexity at the network level, both from a functional and structural point of view [Clayden 2013; Silvestri et al., 2013]. For example, it would be interesting to conduct a psychophysiological interactions (PPI) analyses [Friston et al., 1997] using M1 (or any other key region affected by GF) as the physiological factor and GF as the (multivariate) psychological factor. The keynotes of the distributed network could then be used as regions of interests in dynamic casual modelling (DCM) [Friston et al., 2003]. In this context, the effects of using DH and NDH could be modelled in terms of increases (or decreases) in connectivity due to the task itself or its execution complexity.

## CONCLUSIONS

This study investigated whole brain fMRI activations using the DH and NDH to perform a motor task, with the aim of increasing our understanding of shared and specific functional networks and their dependency upon task and executive complexity. It is evident that a linear increase in the applied GF is supported by an increased activation of motor regions in the left hemisphere, indicative of increased neuronal processing with increased task complexity; on the other hand, the right hemisphere is extensively recruited when adding complexity of task execution by using the NDH. Also, when using the DH, bilateral M1 activations were detected, suggesting a role for the right M1 in performing visuomotor transformation and interhemispheric connections. When using the NDH, activations were seen in different cortical and cerebellar areas, indicating that the NDH can trigger widespread activations that overlap with corresponding DH one, hence engaging the dominant hemisphere in controlling its visually guided movement. Overall, when task complexity is contextualised by execution complexity, several areas are called into action ranging from sensory to more cognitive and associative areas, some activated unilaterally and some activated bilaterally, especially when performing the task with the NDH. These results are not only interesting to understand the functional and structural basis of motor networks, but can have important implications for the design and interpretation of motor studies that aim to characterise abnormal responses in patient groups.

## Supporting information

Supporting Information Figure 1a

Supporting Information Figure 1b

Supporting Information Tables 1‐2ab

Supporting Information Tables 3ab

Supporting Information Tables 4‐9
