# Supplementary figures and images for: Differential involvement of cortical and cerebellar areas using dominant and nondominant hands: An FMRI study
Source: Hum Brain Mapp. 2015 Sep 29;36(12):5079–100. doi: 10.1002/hbm.22997 (PMC4737094; doi:10.1002/hbm.22997)

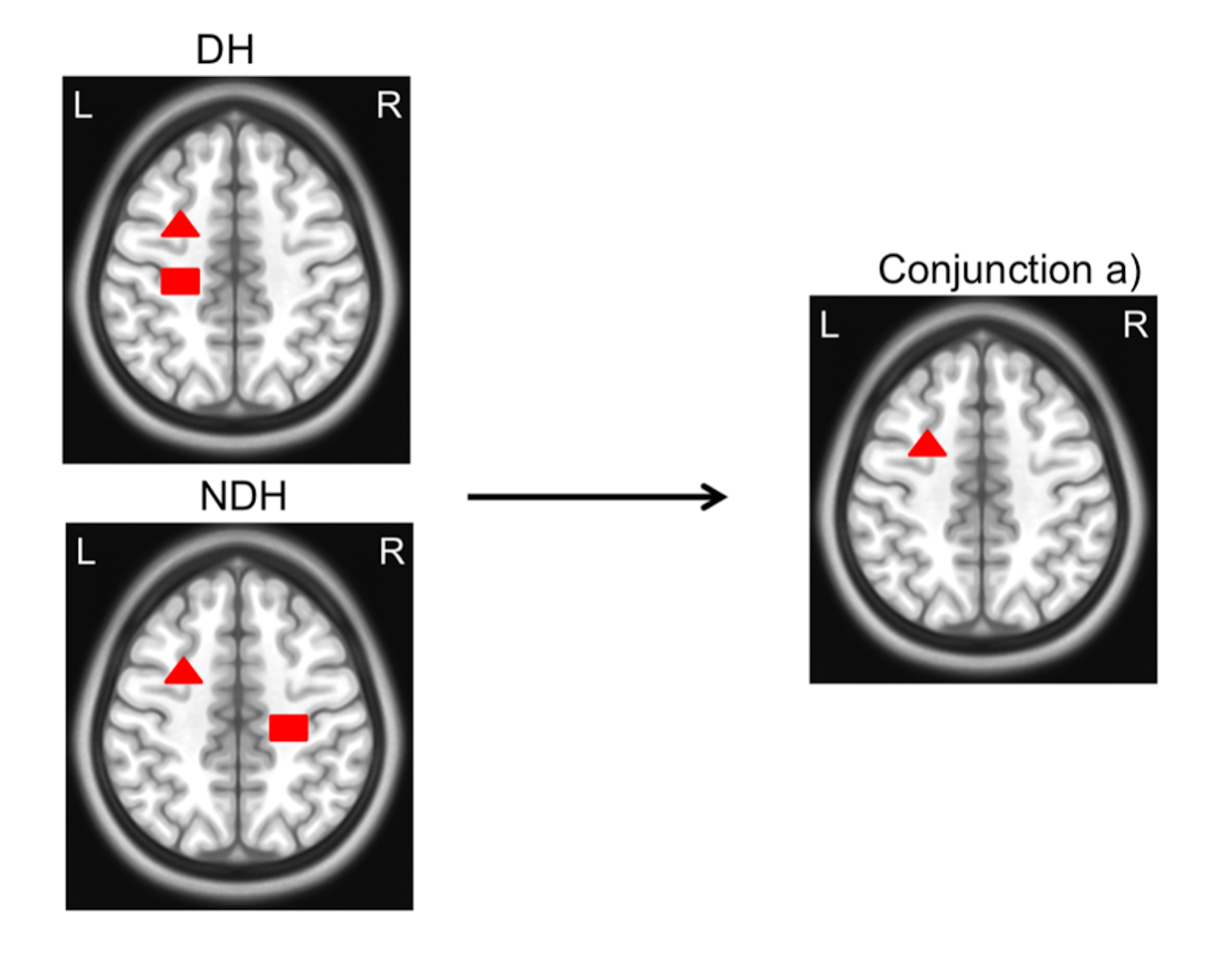

Supplement: Supplementary file 1 — Supporting Information Figure 1a [file HBM-36-5079-s001.tiff]

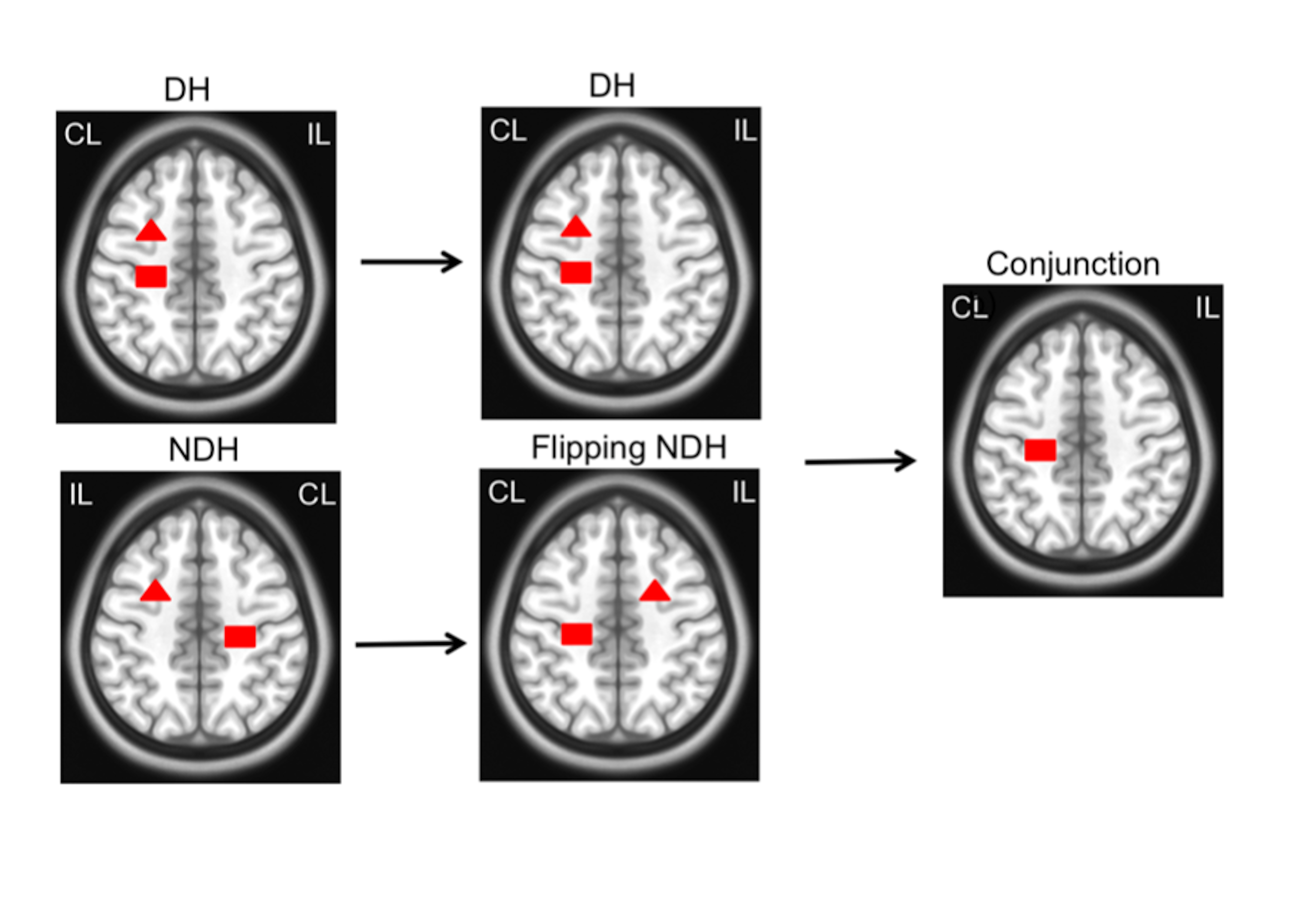

Supplement: Supplementary file 2 — Supporting Information Figure 1b [file HBM-36-5079-s002.tiff]
