# Supplementary material for: Differential involvement of cortical and cerebellar areas using dominant and nondominant hands: An FMRI study
Source: Hum Brain Mapp. 2015 Sep 29;36(12):5079–100. doi: 10.1002/hbm.22997 (PMC4737094; doi:10.1002/hbm.22997)
Supplement: Supplementary file 3 — Supporting Information Tables 1‐2ab [file HBM-36-5079-s003.docx]

| **Table.1 Grip force task performance of the DH and NDH, showing the average (±SD) MVC (%) and duration (s) of all 14 subjects.** | | | |
| --- | --- | --- | --- |
|  | **20 %** | **40 %** | **60 %** |
| **A.DH** |  |  |  |
| **MVC %** | 21.55 ± 3.28 | 39.46 ± 1.29 | 58.11 ± 1.42 |
| **Duration (s)** | 3.04 ± 0.219 | 3.15 ± 0.143 | 3.23 ± 0.225 |
| **B.NDH** |  |  |  |
| **MVC %** | 22.32 ± 2.99 | 39.17 ± 1.35 | 58.39 ± 1.19 |
| **Duration (s)** | 3.14 ± 0.0795 | 3.22 ± 0.0986 | 3.33 ± 1.19 |

| **Table 2.a** **(RFX1)** The main effect of movement (all forces versus baseline) using the DH. In this case R (right) hemisphere = Ipsilateral (IL) side. L (left) hemisphere = Contralateral (CL) side | | | | | | | | | |
| --- | --- | --- | --- | --- | --- | --- | --- | --- | --- |
| **CN** | **Ke** | **T** | **X** | **Y** | **Z** | **Regions** | **BA/Loc (%)** | | **Side** |
|  |  |  |  |  |  |  | **Top** | **Others** |  |
| 2 | 140 | 9.31 | 56 | 4 | 34 | Precentral Gyrus | 6 (40) | 44 (10) | R |
| 2 | 140 | 5.84 | 48 | -1 | 46 |  | 6 (40) |  | R |
| 4 | 153 | 5.52 | 9 | -4 | 67 | Supplementary motor area | 6 (80) |  | R |
| 5 | 35 | 6.87 | 45 | -1 | 7 | Insula Lobe | OP 3 (10) |  | R |
| 1 | 149 | 10.06 | 66 | -16 | 28 | SupraMarginal Gyrus | OP 4 (30) | IPC (PFt) (30); IPC (PF); OP 1; 1; 2; 3b (10) | R |
| 1 | 149 | 8.18 | 63 | -25 | 31 |  | IPC (PF) (60) | OP 1; IPC (PFt) (30); OP 4 (10) | R |
| 2 | 140 | 7.15 | 54 | 8 | 19 | Inferior Frontal Gyrus p. Opercularis | 44 (40) |  | R |
| 3 | 489 | 8.78 | 39 | -58 | -20 | Fusiform Gyrus |  |  | R |
| 3 | 489 | 6.90 | 33 | -70 | -14 |  | hOC5 (V4) (50) |  | R |
| 3 | 489 | 7.96 | 48 | -61 | -2 | Middle Temporal Gyrus | hOC5 (V5) (20) |  | R |
| 3 | 489 | 6.75 | 33 | -85 | -5 | Inferior Occipital Gyrus | hOC5 (V4) (30) | hOC3v (V3v) (20) | R |
| 3 | 489 | 6.61 | 39 | -73 | -5 |  | hOC5 (V4) (10) |  | R |
| 5 | 35 | 5.77 | 54 | 5 | 7 | Rolandic Operculum | 44 (40) | OP 4 (20) | R |
| 6 | 56 | 6.45 | 27 | -61 | 49 | Angular Gyrus | hIP3 (30) | SPL (7A) (20); SPL (7P); hIP1 (10) | R |
| 3 | 489 | 7.39 | 18 | -55 | -23 | Cerebellum | VI (Hem) (90) | V (9) | R |
| 7 | 187 | 7.84 | 21 | -46 | -44 |  |  |  | R |
| 7 | 187 | 7.54 | 18 | -76 | -47 |  | VIIb (Hem) (81) | VIIa Crus II (Hem) (16) | R |
| 7 | 187 | 6.02 | 12 | -43 | -44 |  | IX (Hem) (6) |  | R |
|  |  |  |  |  |  |  |  |  |  |
| 8 | 612 | 8.60 | -45 | -31 | 49 | Postcentral Gyrus | 2 (70) | IPC (PFt); 3b (20) | L |
| 8 | 612 | 8.48 | -36 | -34 | 49 |  | 2 (50) | 3b; 4p; 3a (30); IPC (PFt) (20); SPL (5L) (10) | L |
| 8 | 612 | 8.08 | -54 | -25 | 58 |  |  |  | L |
| 8 | 612 | 8.07 | -42 | -22 | 58 |  | 6 (40) | 4a (40); 3b (30); 1; 2 (20) | L |
| 8 | 612 | 7.51 | -36 | -28 | 67 |  | 6 (50) | 4a (50); 1 (30); 4p (10) | L |
| 8 | 612 | 5.71 | -36 | -10 | 64 |  | 6 (60) |  | L |
| 10 | 153 | 7.64 | 0 | -4 | 52 | Supplementary motor area | 6 (70) |  | L |
| 10 | 153 | 6.24 | -3 | -10 | 61 |  | 6 (90) |  | L |
| 8 | 612 | 8.51 | -60 | -22 | 34 | SupraMarginal Gyrus | IPC (PFt) (70) | IPC (Pfop) (30); IPC (PF); OP 4; 2 (20) | L |
| 8 | 612 | 7.94 | -57 | -22 | 43 |  | 1 (50) | 2 (50); IPC (PFt); IPC (PF) | L |
| 8 | 612 | 8.16 | -39 | -49 | 58 | Inferior Parietal Lobule | SPL (7PC) (60) | hIP3 (30); IPC (PF); 1; 2; IPC (PFm); SPL (7A) (20) | L |
| 9 | 32 | 8.07 | -42 | -4 | 16 | Rolandic Operculum | OP 3 (20) |  | L |
| 11 | 117 | 6.88 | -39 | -73 | -2 | Middle Occipital Gyrus | hOC5 (V5) (30) |  | L |
| 11 | 117 | 6.68 | -48 | -73 | 1 |  | hOC5 (V5) (20) |  | L |
| 11 | 117 | 6.69 | -48 | -67 | -11 | Inferior Occipital Gyrus |  |  | L |
| 12 | 37 | 6.12 | -36 | -55 | -23 | Cerebellum | VI (Hem) (60) | VIIa Crus I (Hem) (2) | L |
| 12 | 37 | 5.54 | -27 | -58 | -29 |  | VI (Hem) (99) |  | L |
| For all tables, The labeled column (BA/Loc (%)) shows the probability (%) of these voxels to be located in the respective Brodmann areas (BA) or specified location (Loc) according to the cytoarchitectonic maps. CN= Cluster number; KE= number of voxels in a cluster; T= T-value at the voxel level. | | | | | | | | | |

| **Table 2.b (RFX1)** The main effect of movement (all forces versus baseline) using the NDH. In this case R (right) hemisphere = Contralateral (CL) side. L (left) hemisphere = Ipsilateral (IL) side. | | | | | | | | | |
| --- | --- | --- | --- | --- | --- | --- | --- | --- | --- |
| **CN** | **Ke** | **T** | **X** | **Y** | **Z** | **Regions** | **BA/Loc (%)** | | **Side** |
|  |  |  |  |  |  |  | **Top** | **Others** |  |
| 2 | 947 | 7.73 | 30 | -19 | 46 | Precentral Gyrus | 4p (30) | 6 (20); 3a (10) | R |
| 2 | 947 | 6.31 | 39 | -13 | 67 |  |  |  | R |
| 3 | 73 | 7.10 | 54 | 5 | 28 |  | 44 (20) | 6 (10) | R |
| 2 | 947 | 12.47 | 48 | -25 | 55 | Postcentral Gyrus | 1 (100) | 2 (60); IPC (PFt) (10) | R |
| 2 | 947 | 10.21 | 57 | -25 | 55 |  |  |  | R |
| 2 | 947 | 9.68 | 33 | -34 | 49 |  | 3b (50) | 3a (40); 2 (30); 4p (20); hIP3 (10) | R |
| 2 | 947 | 7.23 | 42 | -40 | 61 |  | 1 (100) | 2 (50) | R |
| 2 | 947 | 6.91 | 48 | -22 | 34 |  | IPC (PFt) (40) | IPC (PFop) (10); 2 (10); 3b (10) | R |
| 5 | 90 | 6.59 | 6 | -4 | 55 | Supplementary motor area | 6 (80) |  | R |
| 4 | 110 | 8.33 | 33 | -55 | 67 | Superior Parietal Lobule | SPL (7A) (30) | SPL (7PC) (30); hIP3 (10) | R |
| 1 | 1739 | 16.07 | 36 | -85 | -11 | Inferior Occipital Gyrus | hOC4v (V4) (60) | hOC3v (V3v) (40) | R |
| 1 | 1739 | 10.15 | 36 | -64 | -11 | Fusiform Gyrus | hOC4v (V4) (10) |  | R |
| 1 | 1739 | 10.09 | 42 | -79 | 13 | Middle Occipital Gyrus |  |  | R |
| 1 | 1739 | 9.97 | 42 | -67 | 4 |  | hOC5 (V5) (20) |  | R |
| 1 | 1739 | 6.90 | 36 | -88 | 13 |  |  |  | R |
| 1 | 1739 | 6.23 | 33 | -70 | 22 |  |  |  | R |
| 1 | 1739 | 9.88 | 48 | -61 | -17 | Inferior Temporal Gyrus |  |  | R |
| 1 | 1739 | 9.76 | 48 | -58 | -8 |  | hOC5 (V5) (10) |  | R |
| 2 | 947 | 8.49 | 66 | -25 | 40 | SupraMarginal Gyrus |  |  | R |
| 2 | 947 | 6.76 | 66 | -19 | 22 |  | IPC (PFop) (50) | OP 1 (40); OP 4 (40); IPC (PFt); 3b (10) | R |
| 4 | 110 | 6.73 | 30 | -58 | 49 | Angular Gyrus | hIP3 (50) | hIP1 (10) | R |
| 1 | 1739 | 6.21 | 15 | -73 | -23 | Cerebellum | VI (Hem) (93) | VIIa Crus I (Hem) (5) | R |
| 1 | 1739 | 6.00 | 21 | -64 | -23 |  | VI (Hem) (95) | VIIa Crus I (Hem) (5) | R |
| 1 | 1739 | 11.13 | 12 | -82 | -47 |  | VIIa Crus II (Hem) (59) | VIIb (Hem) (41) | R |
|  |  |  |  |  |  |  |  |  |  |
| 7 | 27 | 7.95 | -48 | -31 | 37 | Inferior Parietal Lobule | IPC (PFt) (50) | 2 (30); hIP2 (30) | L |
| 8 | 22 | 5.79 | -36 | -43 | 52 |  | 2 (30) | hIP3; SPL (7PC) (30); SPL (5L) (10) | L |
| 8 | 22 | 6.04 | -39 | -49 | 61 | Superior Parietal Lobule | SPL (7PC) (50) | SPL (7A) (30); 1; 2; hIP3 (20) | L |
| 1 | 1739 | 10.26 | -44 | -73 | -8 | Inferior Occipital Gyrus | hOC5 (V5) (10) |  | L |
| 1 | 1739 | 8.01 | -39 | -55 | -17 | Fusiform Gyrus |  |  | L |
| 1 | 1739 | 7.29 | -39 | -82 | 7 | Middle Occipital Gyrus |  |  | L |
| 1 | 1739 | 7.04 | -36 | -91 | 10 |  | hOC3v (V3v) (20) |  | L |
| 1 | 1739 | 6.38 | -39 | -64 | 4 |  | hOC5 (V5) (10) |  | L |
| 1 | 1739 | 6.10 | -30 | -97 | -2 |  | hOC3v (V3v) (20) | 17 (20); 18 (10) | L |
| 6 | 23 | 5.52 | -32 | -72 | 28 |  |  |  | L |
| 1 | 1739 | 8.07 | -18 | -52 | -17 | Cerebellum | VI (Hem) (85) | V (15) | L |
| 1 | 1739 | 5.95 | -18 | -73 | -23 |  | VI (Hem) (89) | VIIa Crus I (Hem) (11) | L |
| 1 | 1739 | 5.88 | -18 | -58 | -41 |  |  |  | L |
| 1 | 1739 | 7.81 | -12 | -73 | -47 |  | VIIb (Hem) (51) | VIIIa (Hem) (43) | L |
| 1 | 1739 | 6.54 | -6 | -73 | -20 |  | VI (Hem) (85) | V (4) | L |
