# Supplementary material for: Differential involvement of cortical and cerebellar areas using dominant and nondominant hands: An FMRI study
Source: Hum Brain Mapp. 2015 Sep 29;36(12):5079–100. doi: 10.1002/hbm.22997 (PMC4737094; doi:10.1002/hbm.22997)
Supplement: Supplementary file 4 — Supporting Information Tables 3ab [file HBM-36-5079-s004.docx]

| **Table 3.a** **(RFX2)** The effect at each GF level using the DH versus rest (compared using a one sample t-test) | | | | | | | | |  |
| --- | --- | --- | --- | --- | --- | --- | --- | --- | --- |
| CN | Ke | T | X | Y | Z | Regions | BA/Loc (%) |  | Side |
|  |  |  |  |  |  |  | Top | Others |  |
|  | GF of 20% | | | | | | | | |
| 1 | 143 | 8.16 | 56 | 5 | 34 | Precentral Gyrus | 6 (40) | 44 (10) | R |
| 1 | 143 | 5.79 | 48 | -1 | 49 |  | 6 (30) |  | R |
| 2 | 90 | 7.73 | 27 | -58 | 49 | Superior Parietal Lobule | hIP1 (20) | hIP3; SPL (7A) (20) | R |
| 3 | 157 | 9.08 | 63 | -16 | 25 | SupraMarginal Gyrus | IPC (Pfop) (30) | OP 4 (30); IPC (PF); OP 1; 3b; OP 3 (10) | R |
| 3 | 157 | 7.11 | 63 | -28 | 40 |  | IPC (PF) (80) | IPC (PFt) (30); OP 1 (20); IPC (Pfop); 2 (10) | R |
| 3 | 157 | 6.27 | 51 | -34 | 37 |  | IPC (PFcm) (30) | hIP2 (30); IPC (PFm) (10) | R |
| 1 | 143 | 6.70 | 54 | 8 | 19 | Inferior Frontal Gyrus p. Opercularis | 44 (40) |  | R |
| 1 | 143 | 6.66 | 57 | 14 | 1 |  | 44 (40) | 45 (10) | R |
| 4 | 325 | 7.72 | 39 | -58 | -20 | Fusiform Gyrus |  |  | R |
| 4 | 325 | 7.62 | 48 | -58 | -2 | Middle Temporal Gyrus |  |  | R |
| 4 | 325 | 6.18 | 42 | -70 | 1 |  | hOC4v (V5) (20) |  | R |
| 4 | 325 | 5.91 | 36 | -82 | -5 | Inferior Occipital Gyrus | hOC4v (V4) (20) |  | R |
| 4 | 325 | 5.55 | 39 | -79 | 13 | Middle Occipital Gyrus |  |  | R |
| 4 | 325 | 6.68 | 27 | -52 | -29 | Cerebellum | VI (Hem) (85) |  | R |
| 4 | 325 | 6.43 | 24 | -64 | -20 |  | VI (Hem) (89) | VIIa Crus I (Hem) (2) | R |
| 4 | 325 | 6.39 | 18 | -55 | -20 |  | VI (Hem) (86) | V (14) | R |
|  |  |  |  |  |  |  |  |  |  |
| 5 | 478 | 8.69 | -36 | -34 | 49 | Postcentral Gyrus | 2 (50) | 3b; 4p; 3a (30); IPC (PFt) (20); SPL (5L) (10) | L |
| 5 | 478 | 7.80 | -39 | -19 | 52 |  | 4p (60) | 3b; 4a (40); 6 (20) | L |
| 5 | 478 | 7.15 | -57 | -19 | 43 |  | 1 (50) | 2 (40); IPC (PFt) (20); IPC (PF) (10) | L |
| 5 | 478 | 6.36 | -57 | -19 | 34 |  | IPC (PFt) (50) |  | L |
| 6 | 88 | 6.82 | 0 | -4 | 52 | Supplementary motor area | 6 (70) |  | L |
| 6 | 88 | 6.27 | -9 | -4 | 58 |  | 6 (70) |  | L |
| 5 | 478 | 11.08 | -45 | -28 | 46 | Inferior Parietal Lobule | 2 (60) | IPC (PFt) (30); 3b (20); 1(10) | L |
| 5 | 478 | 9.29 | -42 | -46 | 58 |  | 2 (40) | SPL (7PC); 1; hIP3 (20); hIP2 (10) | L |
| 7 | 100 | 6.82 | -48 | -67 | -8 | Inferior Temporal Gyrus | hOC4v (V5) (10) |  | L |
| 7 | 100 | 6.72 | -51 | -70 | 1 | Middle Temporal Gyrus | hOC4v (V5) (10) |  | L |
| GF of 40% | | | | | | | | | |
| 1 | 115 | 5.61 | 48 | -1 | 46 | Precentral Gyrus | 6 (40) |  | R |
| 1 | 115 | 8.05 | 57 | 5 | 34 |  | 6 (40) | 44 (10) | R |
| 2 | 48 | 6.06 | 30 | -61 | 52 | Superior Parietal Lobule | SPL (7A) (50) | hIP3 (40); SPL (7PC); hIP1 (10) | R |
| 2 | 48 | 5.84 | 30 | -61 | 61 |  | SPL (7A) (70) | SPL (7PC); hIP3 (20); SPL (5L); IPC (Pga) (10) | R |
| 3 | 72 | 8.12 | 66 | -16 | 28 | SupraMarginal Gyrus | OP 4 (30) | IPC (PFt) (30); IPC (PF); OP 1; 1; 2; 3b (10) | R |
| 3 | 72 | 6.58 | 66 | -25 | 40 |  |  |  | R |
| 3 | 72 | 6.03 | 57 | -22 | 28 |  | IPC (PFop) (50) | IPC (PFt) (40); OP 1; OP 4; IPC (PFcm); 3b; OP 3 (10) | R |
| 1 | 115 | 6.56 | 51 | 7 | 19 | Inferior Frontal Gyrus p. Opercularis | 44 (50) |  | R |
| 4 | 742 | 9.38 | 39 | -55 | -20 | Fusiform Gyrus |  |  | R |
| 4 | 742 | 8.57 | 33 | -70 | -14 |  | hOC4v (V4) (50) |  | R |
| 4 | 742 | 8.26 | 51 | -61 | -2 | Middle Temporal Gyrus | hOC4v (V5) (30) |  | R |
| 4 | 742 | 6.35 | 30 | -88 | 10 | Middle Occipital Gyrus |  |  | R |
| 4 | 742 | 7.26 | 39 | -79 | 13 |  |  |  | R |
| 4 | 742 | 6.44 | 36 | -82 | -5 | Inferior Occipital Gyrus | hOC4v (V4) (20) |  | R |
| 4 | 742 | 6.29 | 9 | -73 | -35 | Cerebellum | VIIb (Hem) (57) | VIIIa (Hem) (20); VIIa Crus II (Hem) (8); VIIb (Vermis) (2) | R |
| 4 | 742 | 6.09 | 15 | -79 | -44 |  | VIIa Crus II (Hem) (64) | VIIb (Hem) (18) | R |
| 4 | 742 | 7.76 | 21 | -46 | -44 |  |  |  | R |
| 4 | 742 | 7.57 | 18 | -55 | -23 |  | VI (Hem) (90) | V (90) | R |
|  |  |  |  |  |  |  |  |  |  |
| 5 | 472 | 6.72 | -36 | -28 | 64 | Precentral Gyrus | 4a (50) | 6 (30); 1 (20); 4p (20); 2; 3b (10) | L |
| 5 | 472 | 8.54 | -36 | -34 | 49 | Postcentral Gyrus | 2 (50) | 3b; 4p ; 3a (30); IPC (PFt) (20); SPL (5L) (10) | L |
| 5 | 472 | 7.97 | -39 | -19 | 52 |  | 4p (60) | 3b; 4a (40); 6 (20) | L |
| 5 | 472 | 7.79 | -45 | -31 | 49 |  | 2 (70) | IPC (PFt); 3b (20) | L |
| 5 | 472 | 6.93 | -54 | -25 | 58 |  |  |  | L |
| 6 | 119 | 7.27 | -3 | -4 | 52 | Supplementary motor area | 6 (70) |  | L |
| 5 | 472 | 8.31 | -36 | -43 | 52 | Inferior Parietal Lobule | 2 (30) | hIP3; SPL (7PC) (30); SPL (5L) (10) | L |
| 5 | 472 | 7.95 | -39 | -49 | 58 |  | SPL (7PC) (60) | hIP3 (30); IPC (PF); 1; 2; IPC (PFm); SPL (7A) (20) | L |
| 5 | 472 | 6.57 | -57 | -22 | 43 | SupraMarginal Gyrus | 1 (50) | 2 (50); IPC (PFt) (40); IPC (PF) (30) | L |
| 5 | 472 | 6.36 | -60 | -22 | 34 |  | IPC (PFt) (70) | IPC (PFop) (30); IPC (PF); OP 4; 2 (20) | L |
| 7 | 92 | 6.86 | -45 | -76 | -2 | Middle Occipital Gyrus | hOC4v (V5) (30) |  | L |
| 7 | 92 | 6.44 | -48 | -67 | -11 | Inferior Occipital Gyrus |  |  | L |
| 8 | 49 | 6.26 | -36 | -55 | -23 | Cerebellum | VI (Hem) (60) | VIIa Crus I (Hem) (2) | L |
| 8 | 49 | 5.70 | -27 | -61 | -35 |  | VI (Hem) (22) | VIIa Crus I (Hem) (18) | L |
|  |  |  |  | GF of 60% | | | | |  |
| 1 | 144 | 9.57 | 60 | 5 | 34 | Precentral Gyrus | 6 (50) | 44 (10) | R |
| 2 | 135 | 6.90 | 9 | -1 | 55 | Supplementary motor area | 6 (70) |  | R |
| 3 | 121 | 5.98 | 57 | -30 | 52 | Inferior Parietal Lobule | IPC (PFt) (50) | IPC (PF) (40); 2 (20); 1; IPC (PFm); hIP2 (10) | R |
| 3 | 121 | 8.29 | 60 | -28 | 37 | SupraMarginal Gyrus | IPC (PF) (60) | IPC (PFop); IPC (PFt) (30); OP 1 (20) | R |
| 1 | 144 | 6.99 | 45 | -1 | 7 | Insula Lobe | OP 3 (10) |  | R |
| 1 | 144 | 6.86 | 54 | 8 | 22 | Inferior Frontal Gyrus p. Opercularis | 44 (40) |  | R |
| 4 | 316 | 7.48 | 42 | -58 | -17 | Fusiform Gyrus |  |  | R |
| 4 | 316 | 7.18 | 48 | -61 | -2 | Middle Temporal Gyrus | hOC5 (V5) (20) |  | R |
| 4 | 316 | 6.74 | 39 | -73 | -5 | Inferior Occipital Gyrus | hOC5 (V4) (10) |  | R |
| 4 | 316 | 5.70 | 33 | -70 | -14 | Fusiform Gyrus | hOC5 (V4) (50) |  | R |
| 4 | 316 | 6.65 | 18 | -55 | -20 | Cerebellum | VI (Hem) (86) | V (14) | R |
| 4 | 316 | 5.85 | 21 | -67 | -20 |  | VI (Hem) (95) | VIIa Crus I (Hem) (5) | R |
| 5 | 136 | 8.99 | 21 | -67 | -44 |  | VIIb (Hem) (2) | VIIIa (Hem) (1) | R |
|  |  |  |  |  |  |  |  |  |  |
| 6 | 572 | 8.82 | -39 | -28 | 67 | Postcentral Gyrus | 4a (50) | 6 (40); 1 (30) | L |
| 6 | 572 | 8.57 | -42 | -22 | 58 |  | 6 (40) | 4a (40); 3b (30); 1 (20); 2 (10) | L |
| 6 | 572 | 8.36 | -54 | -25 | 58 |  |  |  | L |
| 6 | 572 | 7.31 | -45 | -28 | 49 |  | 2 (70) | 1; 3b (30); IPC (PFt) (20); SPL (5L); 4a ; 3a (10) | L |
| 2 | 135 | 7.14 | -3 | -4 | 52 | Supplementary motor area | 6 (70) |  | L |
| 2 | 135 | 6.18 | -3 | -10 | 61 |  | 6 (90) |  | L |
| 6 | 572 | 6.84 | -36 | -49 | 61 | Superior Parietal Lobule | SPL (7A) (50) | SPL (7PC) (50); 2 (40); 1 (20); SPL (5L); hIP3 (10) | L |
| 6 | 572 | 8.07 | -60 | -22 | 43 | SupraMarginal Gyrus | IPC (PF) (30) | 1; 2 (30); IPC (PFt) (20) | L |
| 6 | 572 | 7.49 | -60 | -22 | 34 |  | IPC (PFt) (70) | IPC (PFop) (30); IPC (PF); OP 4; 2 (20) | L |
| 7 | 44 | 10.28 | -42 | -4 | 13 | Rolandic Operculum | OP 4 (10) | OP 3 (10) | L |
| 8 | 66 | 6.32 | -42 | -67 | -11 | Inferior Occipital Gyrus | hOC5 (V5) (10) |  | L |
| 8 | 66 | 6.21 | -39 | -73 | -2 | Middle Occipital Gyrus | hOC5 (V5) (30) |  | L |
| 8 | 66 | 5.69 | -51 | -73 | 4 |  |  |  | L |

| **Table 3.b (RFX2)** The effect of each GF (using a one sample t test) versus rest using the NDH | | | | | | | | |  |
| --- | --- | --- | --- | --- | --- | --- | --- | --- | --- |
| CN | Ke | T | X | Y | Z | Regions | BA/Loc (%) |  | Side |
|  |  |  |  |  |  |  | Top | Others |  |
|  | GF of 20% | | | | | | | |  |
| 1 | 502 | 10.41 | 48 | -25 | 55 | Postcentral Gyrus | 1 (100) | 2 (60); IPC (PFt) (10) | R |
| 1 | 502 | 10.05 | 57 | -25 | 55 |  |  |  | R |
| 1 | 502 | 7.19 | 45 | -28 | 64 |  | 1 (90) | 3b (10) | R |
| 1 | 502 | 6.78 | 33 | -34 | 49 |  | 3b (50) | 3a (40); 2 (30); 4p (20); hIP3 (10) | R |
| 1 | 502 | 6.64 | 36 | -25 | 46 |  | 4p (70) | 3a (60); 3b (50) | R |
| 2 | 58 | 6.70 | 33 | -58 | 64 | Superior Parietal Lobule | SPL (7A) (60) | SPL (7PC) (40); hIP3 (10) | R |
| 2 | 58 | 6.14 | 27 | -58 | 52 |  | hIP3 (30) | SPL (7A); SPL (7P) (20); SPL (7PC) (10) | R |
| 1 | 502 | 7.49 | 63 | -25 | 37 | SupraMarginal Gyrus | IPC (PFt) (70) | IPC (PF) (60); OP 1 (20) | R |
| 1 | 502 | 6.37 | 48 | -31 | 40 |  | IPC (PFt) (40) | hIP2 (20); SPL (7PC); 2 (10) | R |
| 3 | 57 | 6.62 | 57 | 8 | 25 | Inferior Frontal Gyrus p. Opercularis | 44 (40) |  | R |
| 4 | 567 | 10.51 | 45 | -61 | -8 | Inferior Temporal Gyrus | hOC5 (V5) (10) |  | R |
| 4 | 567 | 10.08 | 38 | -87 | -8 | Inferior Occipital Gyrus | hOC5 (V3v) (20) | hOC4v (V4) (20) | R |
| 4 | 567 | 9.92 | 42 | -70 | 1 | Middle Temporal Gyrus | hOC5 (V5) (20) |  | R |
| 4 | 567 | 7.66 | 36 | -79 | 16 | Middle Occipital Gyrus |  |  | R |
| 5 | 40 | 7.32 | 12 | -79 | -47 | Cerebellum | VIIb (Hem) (50) | VIIa Crus II (Hem) (42); VIIIa (Hem) (5) | R |
|  |  |  |  |  |  |  |  |  |  |
| 6 | 19 | 5.51 | -3 | -4 | 61 | Supplementary motor area | 6 (80) |  | L |
| 7 | 359 | 9.66 | -42 | -76 | -5 | Inferior Occipital Gyrus | hOC5 (V5) (10) |  | L |
| 7 | 359 | 7.71 | -36 | -91 | 10 | Middle Occipital Gyrus | hOC5 (V3v) (20) |  | L |
| 7 | 359 | 7.29 | -30 | -97 | -5 |  | hOC5 (V3v) (50) | 18 (20) | L |
| 7 | 359 | 7.08 | -36 | -94 | 1 |  | hOC5 (V3v) (30) | 18 (10) | L |
| 7 | 359 | 6.99 | -39 | -82 | 7 |  |  |  | L |
| 7 | 359 | 7.01 | -39 | -55 | -17 | Fusiform Gyrus |  |  | L |
| 8 | 22 | 6.49 | -9 | -73 | -44 | Cerebellum | VIIb (Hem) (69) | VIIIa (Hem) (15); VIII (Vermis) (11); VIIa Crus II (Hem) (5) | L |
|  | GF of 40% | | | | | | | | |
| 1 | 818 | 7.44 | 30 | -19 | 46 | Area 4p | 4p (30) | 6 (20); 3a (10) | R |
| 2 | 61 | 6.29 | 57 | 6.5 | 30 | Precentral Gyrus | 44 (20) |  | R |
| 1 | 818 | 10.38 | 48 | -25 | 55 | Postcentral Gyrus | 1 (100) | 2 (60); IPC (PFt) (10) | R |
| 1 | 818 | 8.75 | 30 | -34 | 49 |  | 3a (60) | 2 (40); 3b (40); 4p (30); hIP3 (10) | R |
| 1 | 818 | 7.98 | 36 | -25 | 46 |  | 4p (70) | 3a (60); 3b (50) | R |
| 1 | 818 | 5.66 | 48 | -22 | 34 |  | IPC (PFt) (40) | IPC (PFop) (10); 2 (10); 3b (10) | R |
| 3 | 36 | 6.06 | 3 | -4 | 55 | Supplementary motor area | 6 (90) |  | R |
| 4 | 86 | 7.38 | 33 | -55 | 67 | Superior Parietal Lobule | SPL (7A) (30) | SPL (7PC) (30); hIP3 (10) | R |
| 1 | 818 | 7.23 | 66 | -25 | 40 | SupraMarginal Gyrus |  |  | R |
| 1 | 818 | 6.28 | 66 | -19 | 22 |  | IPC (PFop) (50) | OP 1 (40); OP 4 (40); IPC (PFt) (10); 3b (10) | R |
| 5 | 707 | 17.20 | 36 | -85 | -11 | Inferior Occipital Gyrus | hOC4v (V4) (60) | hOC3v (V3v) (40) | R |
| 5 | 707 | 10.81 | 39 | -64 | -11 |  |  |  | R |
| 5 | 707 | 9.91 | 39 | -73 | -11 |  | hOC4v (V4) (50) |  | R |
| 5 | 707 | 8.09 | 39 | -91 | -2 |  | hOC3v (V3v) (40) | hOC4v (V4) (10) | R |
| 5 | 707 | 7.09 | 36 | -91 | 7 | Middle Occipital Gyrus |  |  | R |
| 5 | 707 | 6.87 | 33 | -70 | 22 |  |  |  | R |
| 5 | 707 | 10.71 | 39 | -76 | 13 |  |  |  | R |
| 5 | 707 | 10.00 | 42 | -67 | 4 |  | hOC5 (V5) (20) |  | R |
| 4 | 86 | 6.45 | 30 | -58 | 52 | Angular Gyrus | hIP3 (40) | SPL (7A) (30); SPL (7PC) (10); hIP1 (10) | R |
| 6 | 678 | 6.04 | 9 | -76 | -20 | Cerebellum | VI (Hem) (95) | VIIa Crus I (Hem) (5) | R |
| 6 | 678 | 5.75 | -18 | -73 | -23 |  | VI (Hem) (89) | VIIa Crus I (Hem) (11) | R |
| 7 | 55 | 8.94 | 12 | -82 | -47 |  | VIIa Crus II (Hem) (59) | VIIb (Hem) (41) | R |
|  |  |  |  |  |  |  |  |  |  |
| 6 | 678 | 10.58 | -48 | -70 | -11 | Inferior Occipital Gyrus |  |  | L |
| 6 | 678 | 7.71 | -39 | -55 | -17 | Fusiform Gyrus |  |  | L |
| 6 | 678 | 7.15 | -30 | -79 | 4 | Middle Occipital Gyrus |  |  | L |
| 6 | 678 | 7.03 | -39 | -88 | 10 |  | hOC3v (V3v) (10) |  | L |
| 6 | 678 | 6.21 | -30 | -97 | -2 |  | hOC3v (V3v) (20) | 17 (20); 18 (10) | L |
| 6 | 678 | 5.84 | -39 | -64 | 4 |  | hOC5 (V5) (10) |  | L |
| 8 | 64 | 7.47 | -12 | -73 | -44 | Cerebellum | VIIb (Hem) (66) | VIIIa (Hem) (12); VIIa Crus II (Hem) (5) | L |
| 6 | 678 | 7.71 | -18 | -52 | -17 |  | VI (Hem) (85) | V (15) | L |
| 6 | 678 | 7.00 | -6 | -73 | -17 |  | VI (Hem) (98) |  | L |
|  | | |  | |  | GF of 60% | |  |  |
| 1 | 1391 | 7.61 | 39 | -13 | 67 | Precentral Gyrus |  |  | R |
| 1 | 1391 | 7.37 | 42 | -10 | 58 |  | 6 (60) |  | R |
| 1 | 1391 | 5.93 | 27 | -13 | 67 |  | 6 (70) |  | R |
| 2 | 59 | 8.40 | 60 | 5 | 31 |  | 6 (30) | 44 (10) | R |
| 1 | 1391 | 14.31 | 48 | -25 | 52 | Postcentral Gyrus | 1 (80) | 2 (70); 3b (20); IPC (PFt) (10) | R |
| 1 | 1391 | 12.26 | 33 | -34 | 49 |  | 3b (50) | 3a (40); 2 (30); 4p (20); hIP3 (10) | R |
| 3 | 137 | 7.48 | 3 | -4 | 52 | Supplementary motor area | 6 (90) |  | R |
| 1 | 1391 | 10.60 | 33 | -55 | 67 | Superior Parietal Lobule | SPL (7A) (30) | SPL (7PC) (30); hIP3 (10) | R |
| 1 | 1391 | 6.15 | 18 | -67 | 55 |  | SPL (7P) (60) | SPL (7A) (20) | R |
| 4 | 20 | 6.78 | 15 | -31 | 1 | Thalamus | Hipp (FD) (20) |  | R |
| 5 | 32 | 6.75 | 36 | -4 | 16 | Insula Lobe | OP 3 (30) | OP 4 (10) | R |
| 1 | 1391 | 9.69 | 63 | -25 | 40 | SupraMarginal Gyrus | IPC (PF) (60) | IPC (PFt) (60); OP 1 ; 2 (20); 1 (10) | R |
| 1 | 1391 | 8.31 | 48 | -25 | 34 |  | IPC (PFt) (30) | OP 1; IPC (PFop); 2 (10) | R |
| 1 | 1391 | 6.49 | 66 | -19 | 22 |  | IPC (PFop) (50) | OP 1; OP 4 (40); IPC (PFt); 3b (10) | R |
| 1 | 1391 | 5.21 | 54 | -37 | 25 |  | IPC (PFcm) (60) | IPC (PFm) (30) | R |
| 6 | 852 | 14.54 | 36 | -88 | -11 | Inferior Occipital Gyrus | hOC3v (V3v) (40) | 18; hOC4v (V4) (30) | R |
| 6 | 852 | 11.09 | 42 | -79 | 13 | Middle Occipital Gyrus |  |  | R |
| 6 | 852 | 7.25 | 36 | -91 | 7 |  |  |  | R |
| 6 | 852 | 7.07 | 36 | -79 | 4 |  |  |  | R |
| 6 | 852 | 9.34 | 42 | -67 | 4 |  | hOCV4v (V4) (20) |  | R |
| 6 | 852 | 8.89 | 48 | -58 | -5 | Inferior Temporal Gyrus |  |  | R |
| 6 | 852 | 9.66 | 48 | -61 | -17 |  |  |  | R |
| 6 | 852 | 9.45 | 45 | -73 | -8 |  |  |  | R |
| 6 | 852 | 9.34 | 39 | -58 | -20 | Fusiform Gyrus |  |  | R |
| 6 | 852 | 9.06 | 36 | -64 | -14 |  |  |  | R |
| 6 | 852 | 7.57 | 45 | -82 | -2 | Inferior Occipital Gyrus | hOCV4v (V4) (20) |  | R |
| 6 | 852 | 6.04 | 51 | -43 | 1 | Middle Temporal Gyrus |  |  | R |
| 1 | 1391 | 7.00 | 30 | -58 | 49 | Angular Gyrus | hIP3 (50) | hIP1 (10) | R |
| 5 | 32 | 6.06 | 45 | -1 | 10 | Rolandic Operculum | OP 4 (10) | OP 3 (10) | R |
| 7 | 1142 | 9.71 | 12 | -76 | -47 | Cerebellum | VIIb (Hem) (68) | VIIIa (Hem) (20); VIIa Crus II (Hem) (6) | R |
| 7 | 1142 | 7.03 | 24 | -67 | -41 |  | VIIa Crus II (Hem) (18) | VIIa Crus I (Hem) (12) | R |
| 6 | 852 | 6.96 | 18 | -67 | -23 |  | VI (Hem) (94) | VIIa Crus I (Hem) (5) | R |
|  |  |  |  |  |  |  |  |  |  |
| 8 | 51 | 8.34 | -48 | -31 | 37 | Inferior Parietal Lobule | IPC (PFt) (50) | 2 (30); hIP2 (30) | L |
| 9 | 48 | 7.39 | -36 | -43 | 52 |  | 2 (30) | hIP3; SPL (7PC) (30); SPL (5L) (10) | L |
| 8 | 51 | 5.68 | -60 | -25 | 40 | SupraMarginal Gyrus | IPC (PF) (40) | IPC (PFt) (40); 2 (30); 1 (10) | L |
| 7 | 1142 | 10.55 | -48 | -70 | -11 | Inferior Occipital Gyrus |  |  | L |
| 7 | 1142 | 7.19 | -39 | -58 | -14 | Fusiform Gyrus |  |  | L |
| 7 | 1142 | 6.72 | -39 | -79 | 7 | Middle Occipital Gyrus | hOC5 (V5) (10) |  | L |
| 7 | 1142 | 6.30 | -39 | -91 | 10 |  |  |  | L |
| 10 | 55 | 5.87 | -33 | -70 | 22 |  |  |  | L |
| 10 | 55 | 5.63 | -33 | -73 | 31 |  | IPC (PGp) (10) | IPC (PGa) (10) | L |
| 7 | 1142 | 6.24 | -3 | -70 | -17 | Cerebellum | VI (Vermis) (60) | VI (Hem) (27); V (7) | L |
| 7 | 1142 | 8.74 | -21 | -61 | -23 |  | VI (Hem) (98) |  | L |
| 7 | 1142 | 8.53 | -18 | -52 | -17 |  | VI (Hem) (85) | V (15) | L |
| 7 | 1142 | 7.42 | -21 | -58 | -44 |  | VIIIb (Hem) (6) |  | L |
| 7 | 1142 | 7.15 | -33 | -55 | -23 |  | VI (Hem) (87) | VIIa Crus I (Hem) (4) | L |
