# Supplementary material for: Differential involvement of cortical and cerebellar areas using dominant and nondominant hands: An FMRI study
Source: Hum Brain Mapp. 2015 Sep 29;36(12):5079–100. doi: 10.1002/hbm.22997 (PMC4737094; doi:10.1002/hbm.22997)
Supplement: Supplementary file 5 — Supporting Information Tables 4‐9 [file HBM-36-5079-s005.docx]

| **Table 4.b** (RFX3) Activated regions using one sample t test at linear response using the non-dominant (left) hand | | | | | | | | | |
| --- | --- | --- | --- | --- | --- | --- | --- | --- | --- |
| CN | ke | T | X | Y | Z | Region | BA/Loc (5) |  | Side |
|  |  |  |  |  |  |  | Top | Others |  |
| 1 | 539 | 12.34 | 36 | -24 | 49 | Postcentral Gyrus | 4p (80) | 4p (80); 3b (30); 4a, 3a (10) | R |
| 1 | 539 | 12.1 | 48 | -19 | 52 |  | 1 (90) | 4a(40); 3b, 6 (20), 2(10) | R |
| 1 | 539 | 10.4 | 39 | -22 | 64 | Precentral Gyrus | 6 (70) | 1,4a (10) | R |
| 1 | 539 | 5.88 | 21 | -34 | 58 |  | 3b (60) | 3b(60); 4p(30); 6,3a (10) | R |
| 1 | 539 | 5.7 | 18 | -16 | 61 |  | 6 (60) |  | R |
| 2 | 370 | 8.1 | 12 | -28 | 4 | Thalamus |  |  | R |
| 2 | 370 | 6.99 | 6 | -42 | 25 | Posterior Cingulum |  |  | R |
| 2 | 370 | 6.58 | 27 | -28 | -5 | Hippocampus |  |  | R |
| 2 | 370 | 5.85 | 12 | -34 | 10 |  |  |  | R |
| 2 | 370 | 6.88 | -6 | -52 | -8 | Cerebellum | V (88) | I-IV (Hem) (10) | L |
| 2 | 370 | 6.79 | -6 | -40 | 1 |  | I-IV (7) |  | L |
| 2 | 370 | 6.38 | -15 | -52 | -17 |  | VI (82) | V (18) | L |
| 2 | 370 | 6.43 | 3 | -46 | -5 | Vermis | I-IV (98) |  |  |

| **Table 4.a (RFX3)** Activated regions using a one sample t-test at Linear effects using the dominant (right) hand | | | | | | | | | |
| --- | --- | --- | --- | --- | --- | --- | --- | --- | --- |
| CN | ke | T | X | Y | Z | Region | BA/Loc (5) |  | Side |
|  |  |  |  |  |  |  | Top | Others |  |
| 1 | 205 | 7.76 | -39 | -28 | 67 | Postcentral Gyrus | 4a (50%) | 6 (40%) | L |
| 1 | 205 | 7.34 | -39 | -25 | 58 |  | 4a, 3b (40%) | 2 (30%) | L |
| 1 | 205 | 6.51 | -33 | -19 | 52 | Precentral Gyrus | 4p (30%) | 6,4a (20%) | L |

| **Table 5.a (RFX4)** Specificity a) of DH vs NDH | | | | | | | | |
| --- | --- | --- | --- | --- | --- | --- | --- | --- |
| **ke** | **T** | **X** | **Y** | **Z** | **Regions** | **BA/Loc (%)** |  | **Side** |
|  |  |  |  |  |  | **Top** | **Others** |  |
| GF of 20% | | |  |  |  |  |  |  |
| 19 | 5.86 | 18 | -52 | -17 | Cerebellum | VI (76) | V (24) | R |
| 19 | 5.70 | 21 | -61 | -11 | Fusiform Gyrus | hOC3v (V3v) (40) | hOC4v (V4) (20); 18 (10) | R |
|  |  |  |  |  |  |  |  |  |
| 215 | 7.71 | -39 | -25 | 58 | Postcentral Gyrus | 3b, 4a (40) | 2 (30); 1, 4p, 6 (20) | L |
| 215 | 6.53 | -54 | -19 | 52 |  | 1 (100) | 2,3b (20); 4a (10) | L |
| GF of 40% | |  |  |  |  |  |  |  |
| 83 | 8.44 | 18 | -52 | -23 | Cerebellum | VI (82) | V (16) | R |
|  |  |  |  |  |  |  |  |  |
| 248 | 8.61 | -36 | -28 | 64 | Precentral Gyrus | 4a (50) | 6 (30); 1,4p (20); 2, 3b (10) | L |
| 248 | 5.97 | -27 | -37 | 67 | Postcentral Gyrus | 1 (50) | 3b (30); 2 (20); | L |
| GF of 60% | |  |  |  |  |  |  |  |
| 82 | 7.09 | 12 | -55 | -11 | Cerebellum | VI (48) | V (45) | R |
| 82 | 7.08 | 18 | -55 | -17 |  | VI (85) | V (15) | R |
|  |  |  |  |  |  |  |  |  |
| 228 | 9.34 | -39 | -28 | 67 | Postcentral Gyrus | 4a (50) | 6 (40); 1 (30) | L |

| **Table 5.b (RFX4)** Specificity a) of NDH vs DH | | | | | | | | |
| --- | --- | --- | --- | --- | --- | --- | --- | --- |
| **ke** | **T** | **X** | **Y** | **Z** | **Regions** | **BA/Loc (%)** |  | **Side** |
|  |  |  |  |  |  | **Top** | **Others** |  |
| GF of 20% | |  |  |  |  |  |  |  |
| 266 | 8.74 | 42 | -28 | 67 | Postcentral Gyrus |  |  | R |
| 266 | 8.30 | 45 | -25 | 58 |  | 1 (90) | 3b (30); 6, 4a (10) | R |
| GF of 40% | | |  |  |  |  |  |  |
| 546 | 10.84 | 45 | -25 | 55 | Postcentral Gyrus | 1, 3b (50) | 2 (20); 4a (10) | R |
| 546 | 8.87 | 54 | -28 | 52 |  | 1 (60) | 2, IPC (PFt) (30); | R |
| 546 | 9.81 | 42 | -25 | 67 | Precentral Gyrus | 6 (60) | 6 (60); 1 (20) | R |
| GF of 60% | |  |  |  |  |  |  |  |
| 472 | 9.87 | 42 | -25 | 64 | Precentral Gyrus | 6 (60) | 1 (50); 4a (20) | R |
| 472 | 8.93 | 33 | -28 | 55 |  | 4p (70) | 3b (30); 4a, 3a (20); 1 (10) | R |
| 472 | 9.49 | 42 | -25 | 55 | Postcentral Gyrus | 3b (70) | 4a (30); 6 (20); 1, 4p (10) | R |
| 472 | 8.67 | 36 | -22 | 46 |  | 4p (90) | 3b (50); 3a (20) | R |
| 472 | 6.27 | 42 | -38 | 66 |  | 1 (100) | 2 (10) | R |
|  |  |  |  |  |  |  |  |  |
| 103 | 10.74 | -18 | -52 | -20 | Cerebellum | VI (85) | V (14) | L |

| **Table 6.a (RFX4)** Lateralization b) of the DH ( DH vs fDH**)** | | | | | | | | |
| --- | --- | --- | --- | --- | --- | --- | --- | --- |
| **ke** | **T** | **X** | **Y** | **Z** | **Regions** | **BA/Loc (%)** |  | **Side** |
|  |  |  |  |  |  | **Top** |  |  |
| GF of 20% | |  |  |  |  |  |  |  |
| 12 | 7.22 | 30 | -61 | 52 | Superior Parietal Lobule | SPL (7A) (50) | hIP3 (40); SPL (7PC) (10) | R |
| 58 | 6.82 | 15 | -58 | -14 | Cerebellum | VI (83) | V (17) | R |
| 58 | 6.27 | 15 | -55 | -26 |  | VI (59) | VI (59); V (18) | R |
|  |  |  |  |  |  |  |  |  |
| 306 | 8.38 | -36 | -28 | 67 | Precentral Gyrus | 6, 4a (50) | 1 (30); 4p (10) | L |
| 306 | 6.39 | -36 | -13 | 58 |  | 6 (60) | 4a (10) | L |
| 306 | 8.25 | -42 | -25 | 58 | Postcentral Gyrus | 1 (40) | 2, 4a (30); 3b (20); 6 (10) | L |
| 306 | 7.16 | -48 | -13 | 55 |  | 1 (30) | 6 (10) | L |
| 306 | 6.69 | -24 | -28 | 70 |  | 6 (30) | 1, 3b (10) | L |
| 58 | 6.35 | 6 | -67 | -20 | Cerebellum | VI (73) | VI (Vermis) (10) |  |
| GF of 40% | |  |  |  |  |  |  |  |
| 12 | 8.56 | 48 | 5 | 19 | Inferior Frontal Gyrus | 44 (40) |  | R |
| 36 | 6.68 | 39 | 41 | 19 | Middle Frontal Gyrus |  |  | R |
| 36 | 6.30 | 36 | 47 | 28 |  |  |  | R |
| 171 | 7.54 | 15 | -55 | -20 | Cerebellum | VI (80) | V (16) | R |
| 171 | 7.34 | 21 | -46 | -26 |  | VI, V (46) |  | R |
| 171 | 6.90 | 27 | -58 | -17 |  | hOC3v (V3v) (10) | VI (3) | R |
|  |  |  |  |  |  |  |  |  |
| 304 | 8.44 | -39 | -28 | 67 | Postcentral Gyrus | 4a (50) | 6 (40); 1 (30) | L |
| 304 | 8.35 | -42 | -25 | 58 |  | 1 (40) | 2, 4a (30); 3b (20); 6 (10) | L |
| 304 | 6.48 | -21 | -28 | 73 |  |  |  | L |
| 11 | 6.61 | -45 | -25 | 19 | Rolandic Operculum | OP 1 (90) |  | L |
| 171 | 7.80 | 6 | -70 | -35 | Cerebellum | VIIIa (Vermis) (69) | VIIb (Vermis) (29) |  |
| 171 | 6.88 | 3 | -70 | -23 |  | VI (Vermis) (66) |  |  |
| 171 | 5.16 | 6 | -55 | -8 |  | V (96) |  |  |
| GF of 60% | |  |  |  |  |  |  |  |
| 10 | 6.67 | 51 | 17 | 34 | Inferior Frontal Gyrus | 44 (60) | 45 (40) | R |
| 18 | 6.43 | 6 | 17 | 37 | Middle Cingulum | 6 (10) |  | R |
| 18 | 5.58 | 6 | 26 | 37 |  |  |  | R |
| 182 | 9.48 | 15 | -58 | -20 | Cerebellum | VI (93) |  | R |
| 182 | 7.56 | 24 | -46 | -26 |  | VI (74) | V (23) | R |
| 15 | 8.39 | 25.5 | -55 | -44 |  |  |  | R |
|  |  |  |  |  |  |  |  |  |
| 300 | 10.52 | -39 | -28 | 64 | Postcentral Gyrus | 4a (60) | 1, 6 (40); 2, 3b (10) | L |
| 300 | 7.66 | -21 | -28 | 73 |  |  |  | L |
| 300 | 7.27 | -21 | -31 | 64 |  | 4a (40) | 4p, 6 (30); 1, 3b (20); 2 (10) | L |
| 32 | 12.06 | 6 | -70 | -38 | Cerebellum | VIIIa (Vermis) (89) |  |  |
| 182 | 6.94 | 3 | -52 | -8 |  | V (50) | I-IV (14) |  |

| **Table 6.b (RFX4)** Lateralization of the NDH ( NDH vs fNDH) | | | | | | | | |
| --- | --- | --- | --- | --- | --- | --- | --- | --- |
| **ke** | **T** | **X** | **Y** | **Z** | **Regions** | **BA/Loc (%)** |  | **Side** |
|  |  |  |  |  |  | **Top** |  |  |
| GF of 20% | |  |  |  |  |  |  |  |
| 271 | 10.82 | 54 | -28 | 52 | Postcentral Gyrus | 1 (60) | 2 (30); IPC (PFt) (30) | R |
| 271 | 8.66 | 45 | -28 | 64 |  | 1 (90) | 3b (10) | R |
| 33 | 7.91 | 60 | -28 | 31 | SupraMarginal Gyrus | IPC (PF) (70) | OP 1 (20); hIP2 (20) | R |
| 14 | 7.60 | 39 | -22 | 22 | Rolandic Operculum | OP 2 (30) | OP 1 (20); OP 3 (10) | R |
| 10 | 6.42 | 30 | 14 | 7 | Putamen |  |  | R |
|  |  |  |  |  |  |  |  |  |
| 41 | 7.53 | -21 | -55 | -17 | Cerebellum | VI (95) | V (5) | L |
| GF of 40% | |  |  |  |  |  |  |  |
| 389 | 11.39 | 51 | -28 | 52 | Postcentral Gyrus | 1 (60) | 2 (50); IPC (PFt) (20) | R |
| 389 | 9.19 | 45 | -28 | 64 |  | 1 (90) | 3b (10) | R |
| 389 | 5.65 | 24 | -37 | 67 |  | 3b (60) | 4p, 4a (30); 6 (20); SPL (7PC) (10) | R |
| 32 | 8.37 | 42 | -22 | 22 | Rolandic Operculum | OP 1 (50) | OP 2, OP 3 (30) | R |
| 21 | 6.68 | 39 | 44 | 22 | Middle Frontal Gyrus |  |  | R |
| 24 | 6.10 | 63 | -28 | 31 | SupraMarginal Gyrus | IPC (PF) (80) |  | R |
| 24 | 5.58 | 60 | -19 | 25 |  | OP 1, IPC (PFop) (30) | 3b (10) | R |
| 24 | 5.49 | 57 | -25 | 19 |  | OP 1 (50) | IPC (PFop) (40) | R |
|  |  |  |  |  |  |  |  |  |
| 70 | 7.08 | -18 | -55 | -20 | Cerebellum | VI (93) |  | L |
| 70 | 6.07 | -9 | -55 | -8 |  | V (64) | VI (36) | L |
| GF of 60% | |  |  |  |  |  |  |  |
| 359 | 9.80 | 51 | -28 | 52 | Postcentral Gyrus | 1 (60) | 2 (50); IPC (PFt) (20) | R |
| 359 | 9.09 | 45 | -25 | 58 |  | 1 (90) | 3b (30); 6, 4a (10) | R |
| 54 | 9.55 | 33 | -19 | 4 | Insula | Insula (Ig1) (60); |  | R |
| 54 | 7.78 | 36 | -13 | 13 |  | OP 3 (50) | OP 2 (40); Insula (Ig2) (10) | R |
| 15 | 10.36 | 27 | -1 | -2 | Pallidum |  | Putamen | R |
| 10 | 6.29 | 60 | -28 | 34 | SupraMarginal Gyrus | IPC (PF) (50) | IPC (PFop) (30) | R |
| 23 | 8.35 | 42 | 41 | 25 | Middle Frontal Gyrus |  |  | R |
| 23 | 7.43 | 36 | 44 | 19 |  |  |  | R |
| 20 | 7.32 | 42 | -22 | 22 | Rolandic Operculum | OP 1 (50) | OP 2, OP 3 (30) | R |
| 11 | 6.92 | 48 | 8 | 22 | Inferior Frontal Gyrus | 44 (60) |  | R |
| 11 | 6.03 | 57 | 11 | 28 |  | 44 (50) | 45, 6 (10) | R |
|  |  |  |  |  |  |  |  |  |
| 14 | 8.04 | -18 | -55 | -5 | Lingual | 18 (30) | 17 (20) | L |
|  | | | | | | | | |
|  | | | | | | | | |

| **Table 7.b (RFX4)** Strength of activation. a) fNDH vs DH | | | | | | | |
| --- | --- | --- | --- | --- | --- | --- | --- |
| **ke** | **T** | **X** | **Y** | **Z** | **Region** | **BA/Loc (%)** | **Side** |
|  |  |  |  |  |  | **Top** |  |
| GF of 20% | |  |  |  |  |  |  |
| NA | NA | NA | NA | NA | NA | NA | NA |
| GF of 40% | |  |  |  |  |  |  |
| 19 | 6.35 | -39 | 44 | 25 | Middle Frontal Gyrus | NA | L |
| GF of 60% | |  |  |  |  |  |  |
| NA | NA | NA | NA | NA | NA | NA | NA |

| **Table 7.a (RFX4)** Strength of activation. a) DH vs fNDH | | | | | | | | |
| --- | --- | --- | --- | --- | --- | --- | --- | --- |
| **ke** | **T** | **X** | **Y** | **Z** | **Region** | **BA/Loc (%)** |  | **Side** |
|  |  |  |  |  |  | **Top** | **Others** |  |
| GF of 20% | |  |  |  |  |  |  |  |
| 16 | 6.71 | 54 | 11 | 4 | Inferior Frontal Gyrus | 44 (50) | 44 (50); 45 (10) | R |
| 21 | 6.47 | 63 | -31 | 31 | SupraMarginal Gyrus | IPC (PF) (90) | IPC (PF) (90) | R |
| 10 | 6.00 | 45 | 38 | 10 | Inferior Frontal Gryus | 45 (20) | 45 (20) | R |
| GF of 40% | |  |  |  |  |  |  |  |
| NA | NA | NA | NA | NA | NA | NA | NA | NA |
| GF of 60% | |  |  |  |  |  |  |  |
| 14 | 6.22 | 57 | 11 | 34 | Precentral Gyrus | 44 (20) | 6 (10) | R |

| **Table 8.a** common activations regions as a result of the conjunction analysis a) at GF of 20% | | | | | | | | |
| --- | --- | --- | --- | --- | --- | --- | --- | --- |
| CN | KE | X | Y | Z | Region | BA/Loc (%) |  |  |
|  |  |  |  |  |  | Top | Others | Side |
| GF of 20% | | | | | | | | |
| 4 | 42 | 51 | 8 | 37 | Precentral | 44 (20) | 6 (10) | R |
| 2 | 12 | 2 | -4 | 58 | Supplementary motor area | 6 (90) |  | R |
| 3 | 37 | 29 | -56 | 48 | Superior parietal lobule | hIP3 (30) | hIP1 (10) | R |
| 3 | 37 | 30 | -60 | 57 |  | SPL (7A) (60) | hIP3 (30); SPL (7P;PC) (10) | R |
| 5 | 79 | 61 | -24 | 32 | SupraMarginal | IPC (PFt) (70) | IPC (PF) (40); OP 1 (20) | R |
| 5 | 79 | 61 | -27 | 42 |  | IPC (PF), (PFt) (60) | 2 (20) | R |
| 5 | 79 | 51 | -33 | 39 |  | IPC (PFt), hIP2 (30) | IPC (PFop) (10) | R |
| 4 | 42 | 56 | 8 | 28 | Inferior Frontal Operc | 44 (20) |  | R |
| 6 | 150 | 38 | -60 | -19 | Fusiform |  |  | R |
| 6 | 150 | 35 | -68 | -16 |  | hOC4v (V4) (30) |  | R |
| 6 | 150 | 47 | -64 | -9 | Inferior Temporal Gyrus | hOC5 (V5) (10) |  | R |
| 6 | 150 | 36 | -82 | -5 | Inferior Occipital Gyrus | hOC4v (V4) (20) |  | R |
| 6 | 150 | 47 | -63 | 0 | Middle Temporal Gyrus | hOC5 (V5) (40) |  | R |
| 6 | 150 | 40 | -74 | 2 | Middle Occipital Gyrus |  |  | R |
| 6 | 150 | 38 | -80 | 12 |  |  |  | R |
| 1 | 11 | 16 | -75 | -47 | Cerebellum | VIIb (69) | VIIIa (Hem) (8) | R |
|  |  |  |  |  |  |  |  |  |
| 7 | 95 | -46 | -67 | -7 | Inferior Temporal Gyrus | hOC5 (V5) (10) |  | L |
| 7 | 95 | -41 | -73 | -5 | Inferior Occipital Gyrus | hOC5 (V5) (30) |  | L |
| 7 | 95 | -47 | -71 | 0 | Middle Occipital Gyrus | hOC5 (V5) (20) |  | L |
| 7 | 95 | -42 | -80 | 2 |  | hOC5 (V5) (10) |  | L |

| **Table 8.b** common activations regions as a result of the conjunction analysis a) at GF of 40% | | | | | | | | | |
| --- | --- | --- | --- | --- | --- | --- | --- | --- | --- |
| CN | KE | | X | Y | Z | Region | BA/Loc (%) |  |  |
|  |  | |  |  |  |  | Top | Others | Side |
| GF of 40% | | | | | | | | | |
| 5 | 52 | 57 | | 7 | 28 | Precentral | 44 (20) |  | R |
| 6 | 58 | 66 | | -16 | 22 | Postcentral | OP 4 (50) | IPC (PFop) (40); 1 (10) | R |
| 7 | 38 | 30 | | -60 | 60 | Superior parietal lobule | SPL (7A) (90) | SPL (7P) (10) | R |
| 6 | 58 | 63 | | -21 | 30 | SupraMarginal | IPC (PFt) (50) | 1,2,3b (10) | R |
| 6 | 58 | 64 | | -26 | 39 |  | IPC (PF) (60) | 2 (10) | R |
| 6 | 58 | 62 | | -27 | 48 |  |  |  | R |
| 7 | 38 | 30 | | -58 | 52 | Angular | hIP3 (40) | SPL (7A) (30); SPL (7PC) (10) | R |
| 2 | 320 | 40 | | -58 | -20 | Fusiform |  |  | R |
| 2 | 320 | 31 | | -62 | -19 |  | VI (Hem) (11) |  | R |
| 2 | 320 | 36 | | -70 | -16 |  | hOC4v (V4) (20) |  | R |
| 2 | 320 | 46 | | -61 | -8 | Inferior Temporal Gyrus | hOC5 (V5) (10) |  | R |
| 2 | 320 | 41 | | -69 | -8 | Inferior Occipital Gyrus | hOC5 (V5) (10) |  | R |
| 2 | 320 | 36 | | -82 | -2 |  | hOC4v (V4) (10) |  | R |
| 2 | 320 | 44 | | -67 | 2 | Middle Temporal Gyrus | hOC5 (V5) (40) |  | R |
| 2 | 320 | 48 | | -60 | 1 |  | hOC5 (V5) (10) |  | R |
| 2 | 320 | 38 | | -77 | 11 | Middle Occipital Gyrus |  |  | R |
| 3 | 14 | 31 | | -88 | 5 |  |  |  | R |
| 4 | 14 | 34 | | -73 | 23 |  |  |  | R |
| 5 | 52 | 57 | | 10 | 15 | Inferior Frontal Operc | 44 (60) |  | R |
| 1 | 15 | 14 | | -76 | -45 | Cerebellum | VIIb (Hem) (53) | VIIa Crus II (Hem) (13) | R |
| 1 | 15 | 6 | | -76 | -38 |  | VIIIa (Vermis) (34) | VIIb (Vermis) (19) | R |
| 1 | 15 | 16 | | -72 | -21 |  | VI (Hem) (95) | VIIa Crus I (Hem) (5) | R |
|  |  |  | |  |  |  |  |  |  |
| 8 | 34 | 1 | | -4 | 55 | Supplementary Motor Area | 6 (90) (L SMA) |  | L |
| 10 | 88 | -45 | | -68 | -9 | Inferior Temporal Gyrus |  |  | L |
| 10 | 88 | -41 | | -76 | -6 | Inferior Occipital Gyrus | hOC5 (V5) (10) |  | L |
| 10 | 88 | -48 | | -72 | 1 | Middle Occipital Gyrus | hOC5 (V5) (20) |  | L |
| 10 | 88 | -43 | | -80 | 3 |  |  |  | L |
| 9 | 31 | -36 | | -55 | -24 | Cerebellum | VI (Hem) (60) | VIIa Crus I (2) | L |

| **Table 8.c** common activations regions as a result of the conjunction analysis a) at GF of 60% | | | | | | | | | |
| --- | --- | --- | --- | --- | --- | --- | --- | --- | --- |
| CN | KE | | X | Y | Z | Region | BA/Loc (%) |  |  |
|  |  | |  |  |  |  | Top | Others | Side |
| GF of 60% | | | | | | | | | |
| 5 | 51 | 57 | | 6 | 29 | Precentral | 44 (20) | 6 (10) | R |
| 6 | 85 | 7 | | -2 | 56 | Supplementary Motor Area | 6 (70) |  | R |
| 4 | 113 | 53 | | -32 | 51 | Inferior Parietal Lobule | IPC (PF) (50) | 2 (20); 1 (10) | R |
| 4 | 113 | 61 | | -22 | 29 | SupraMarginal | IPC (PFop) (50) | 3b (10) | R |
| 4 | 113 | 58 | | -28 | 36 |  | IPC (PF) (4) |  | R |
| 4 | 113 | 64 | | -22 | 39 |  | IPC (PFt) (60) | 2 (40); 1 (10) | R |
| 4 | 113 | 60 | | -27 | 50 |  |  |  | R |
| 2 | 177 | 40 | | -59 | -18 | Fusiform |  |  | R |
| 2 | 177 | 33 | | -64 | -16 |  | hOC4v (V4) (20) |  | R |
| 2 | 177 | 35 | | -72 | -13 |  | hOC4v (V4) (40) |  | R |
| 2 | 177 | 39 | | -72 | -4 |  | hOC5 (V5); hOC4v (V4) (10) |  | R |
| 2 | 177 | 45 | | -63 | -1 | Middle Temporal Gyrus | hOC5 (V5) (30) |  | R |
| 2 | 177 | 49 | | -59 | -8 | Inferior Temporal Gyrus | hOC5 (V5) (10) |  | R |
| 3 | 11 | 45 | | -2 | 9 | Rolandic Oper | OP 3 (20) | OP 4 (10) | R |
| 1 | 50 | 14 | | -73 | -43 | Cerebellum | VIIb (Hem) (53) | VIIIa (Hem) (14) | R |
| 1 | 50 | 21 | | -67 | -43 |  |  |  | R |
| 2 | 177 | 27 | | -59 | -22 |  | VI (96) |  | R |
| 2 | 177 | 34 | | -54 | -22 |  | VI (6) |  | R |
| 2 | 177 | 21 | | -66 | -22 |  | VI (Hem) (95) | VIIa Crus I (Hem) (5) | R |
|  |  |  | |  |  |  |  |  |  |
| 6 | 85 | -2 | | -7 | 55 | Supplementary Motor Area | 6 (70) | 6 (70) | L |
| 6 | 85 | -6 | | -10 | 63 |  | 6 (90) | 6 (90) | L |
| 11 | 30 | -38 | | -48 | 62 | Superior parietal lobule | SPL (7PC) (70) | SPL (7A), 2 (40); 1(20) | L |
| 8 | 10 | -60 | | -25 | 41 | SupraMarginal | IPC (PF) (40) | 2 (30); 1 (10) | L |
| 9 | 11 | -47 | | -30 | 44 | Inferior parietal lobule | 2 (60) | 1 (10) | L |
| 10 | 30 | -37 | | -42 | 54 |  | 2 (40) | SPL (7PC) (30); 3b (10) | L |
| 6 | 85 | 1 | | -1 | 48 | Middle Cingulum | 6 (50) | 6 (50) | L |
| 7 | 64 | -44 | | -67 | -10 | Inferior Occipital Gyrus |  |  | L |
| 7 | 64 | -39 | | -72 | -5 |  | hOC5 (V5) (20) |  | L |
| 7 | 64 | -51 | | -70 | 2 | Middle Temporal Gyrus |  |  | L |
| 7 | 64 | -43 | | -73 | 3 | Middle Occipital Gyrus | hOC5 (V5) (50) |  | L |

| **Table 9.a** common activations regions as a result of the conjunction analysis b) at GF of 20% | | | | | | | | |
| --- | --- | --- | --- | --- | --- | --- | --- | --- |
| CN | KE | X | Y | Z | Regions | BA/Loc (%) |  | Side |
|  |  |  |  |  |  | Top | Others |  |
| GF of 20% | | | | | | | | |
| 2 | 86 | 38 | -56 | -20 | Fusiform |  |  | IL |
| 2 | 86 | 43 | -64 | -16 | Inferior Occipital Gyrus |  |  | IL |
| 2 | 86 | 36 | -82 | -4 |  | hOC4v (V4) ( 10) |  | IL |
| 2 | 86 | 45 | -66 | -1 | Middle Temporal Gyrus | hOC5 (V5) (40) |  | IL |
| 2 | 86 | 40 | -73 | 0 | Middle Occipital Gyrus |  |  | IL |
| 2 | 86 | 39 | -77 | 9 |  |  |  | IL |
| 1 | 15 | 14 | -74 | -46 | Cerebellum | VIIb (Hem) (56) | VIIIa (22) | IL |
|  |  |  |  |  |  |  |  |  |
| 4 | 272 | -41 | -25 | 64 | Precentral | 6 (60) | 1, 4a (30); 2 (10) | CL |
| 4 | 272 | -58 | -21 | 33 | Postcentral | OP 1, OP 4 , 2 (10) |  | CL |
| 4 | 272 | -37 | -30 | 49 |  | 4p (60) | 2 (40); 3b (30); 3a (20) | CL |
| 4 | 272 | -56 | -20 | 51 |  | 1 (90) | 2 (30); 4a (10) | CL |
| 4 | 272 | -41 | -23 | 53 |  | 3b (50) | 4a (40); 2 (30); 1, 4p (20) | CL |
| 4 | 272 | -48 | -20 | 57 |  | 1 (90) | 3b (30) | CL |
| 4 | 272 | -51 | -28 | 57 |  | 1 (90) | 2(30); IPC (PFt) (10) | CL |
| 4 | 272 | -40 | -30 | 57 |  | 1 (70) | 3b (70); 4a (50); 2 (40); 4p (20) | CL |
| 5 | 13 | 0 | -4 | 58 | Supplementary Motor Area | 6 (80) |  | CL |
| 4 | 272 | -60 | -23 | 44 | Inferior parietal lobule | 2 (40) | IPC (PF), 1 (30) | CL |
| 4 | 272 | -47 | -29 | 46 |  | 2 (80) | IPC (PFt) (30); 1 (20) | CL |
| 4 | 272 | -36 | -41 | 50 |  | 2, hIP3 (30) | SPL (7PC) (20) | CL |
| 3 | 97 | -46 | -67 | -6 | Inferior Temporal Gyrus | hOC5 (V5) (10) |  | CL |
| 3 | 97 | -41 | -73 | -5 | Inferior Occipital Gyrus | hOC5 (V5) (30) |  | CL |
| 3 | 97 | -48 | -73 | 1 | Middle Occipital Gyrus | hOC5 (V5) (20) |  | CL |
| 3 | 97 | -41 | -80 | 3 |  | hOC5 (V5) (10) |  | CL |

| **Table 9.b** common activations regions as a result of the conjunction analysis b) at GF of 40% | | | | | | | | |
| --- | --- | --- | --- | --- | --- | --- | --- | --- |
| CN | KE | X | Y | Z | Regions | BA/Loc (%) |  | Side |
|  |  |  |  |  |  | Top | Others |  |
| GF of 40% | | | | | | | | |
| 2 | 325 | 40 | -58 | -19 | Fusiform |  |  | IL |
| 2 | 325 | 38 | -70 | -15 |  | hOC4v (V4) |  | IL |
| 2 | 325 | 43 | -65 | -8 | Inferior Temporal Gyrus | hOC5 (V5) (10) |  | IL |
| 2 | 325 | 39 | -77 | -3 | Inferior Occipital Gyrus |  |  | IL |
| 2 | 325 | 43 | -66 | 0 | Middle Temporal Gyrus | hOC5 (V5) (20) |  | IL |
| 2 | 325 | 37 | -81 | 8 | Middle Occipital Gyrus |  |  | IL |
| 3 | 10 | 31 | -88 | 5 |  |  |  | IL |
| 1 | 32 | 14 | -72 | -44 | Cerebellum | VIIb (53) | VIIIa (13) | IL |
| 2 | 325 | 30 | -56 | -23 |  | VI (100) |  | IL |
| 2 | 325 | 20 | -53 | -24 |  | VI (92) | V (8) | IL |
| 2 | 325 | 18 | -70 | -21 |  | VI (95) | VIIa Crus I (Hem) (5) | IL |
| 2 | 325 | 17 | -58 | -18 |  | VI (95) | V (5) | IL |
|  |  |  |  |  |  |  |  |  |
| 6 | 380 | -38 | -16 | 52 | Precentral | 6, 4a (50) | 3b, 4p (20) | CL |
| 6 | 380 | -42 | -19 | 59 |  | 6 (60) | 4a (40); 1 (30); 3b (20) | CL |
| 6 | 380 | -39 | -27 | 65 |  | 4a (50) | 6 (40); 1 (30) | CL |
| 6 | 380 | -36 | -38 | 51 | Postcentral | 2 (50) | 3b (30); SPL (5L), hIP3 (20) | CL |
| 6 | 380 | -38 | -25 | 50 |  | 3b (60) | 2 (30); 4p, 4a, 3a (30); 1 (10) | CL |
| 6 | 380 | -38 | -30 | 57 |  | 4a (60) | 3b (40); 4p (30); 2 (20) | CL |
| 6 | 380 | -50 | -25 | 55 |  | 1 (90) | 2(30) | CL |
| 6 | 380 | -47 | -14 | 54 |  | 1 (40) | 6 (30); 3b, 4a (20) | CL |
| 6 | 380 | -37 | -42 | 61 |  | 2 (50) | 1 (30); SPL (7PC), 3b (10) | CL |
| 7 | 32 | -1 | -4 | 54 | Supplementary Motor Area | 6 (70) |  | CL |
| 6 | 380 | -47 | -29 | 46 | Inferior parietal lobule | 2 (80) | 1 (20) | CL |
| 6 | 380 | -57 | -22 | 47 |  | 1 (70) | 2 (50) | CL |
| 6 | 380 | -58 | -22 | 34 | SupraMarginal | IPC (PFt) (80) | 2(30); 1 (10) | CL |
|  |  |  |  |  |  |  |  |  |
| 5 | 86 | -45 | -68 | -8 | Inferior Temporal Gyrus | hOC5 (V5) (10) |  | CL |
| 5 | 86 | -41 | -75 | -7 | Inferior Occipital Gyrus | hOC5 (V5) (10) |  | CL |
| 5 | 86 | -44 | -76 | 1 | Middle Occipital Gyrus | hOC5 (V5) (40) |  | CL |
| 5 | 86 | -52 | -70 | 1 | Middle Temporal Gyrus |  |  | CL |
| 4 | 19 | -37 | -55 | -23 | Cerebellum | VI (60) |  | CL |

| **Table 9.c** common activations regions as a result of the conjunction analysis b) at GF of 60% | | | | | | | | |
| --- | --- | --- | --- | --- | --- | --- | --- | --- |
| CN | KE | X | Y | Z | Regions | BA/Loc (%) |  | Side |
|  |  |  |  |  |  | Top | Others |  |
| GF of 60% | | | | | | | | |
| 3 | 15 | 53 | -29 | 36 | SupraMarginal | IPC (PFop), (PFt) (30) | OP 1 (10) | IL |
| 3 | 15 | 61 | -25 | 41 |  | IPC (PFt) (60) | IPC (PF) (50); 2 (20); 1 (10) | IL |
| 2 | 252 | 39 | -57 | -18 | Fusiform |  |  | IL |
| 2 | 252 | 36 | -70 | -13 |  | hOC4v (V4) (50) |  | IL |
| 2 | 252 | 39 | -72 | -4 |  | hOC5 (V5) (10) |  | IL |
| 2 | 252 | 44 | -65 | -2 | Middle Temporal Gyrus | hOC5 (V5) (30) |  | IL |
| 1 | 97 | 15 | -71 | -44 | Cerebellum | VIIIa (37) | VIIb (29) | IL |
| 1 | 97 | 21 | -64 | -44 |  | VIIIa (2) |  | IL |
| 1 | 97 | 24 | -56 | -46 |  |  |  | IL |
| 2 | 252 | 29 | -52 | -23 |  | VI (90 | V (10) | IL |
| 2 | 252 | 21 | -56 | -23 |  | VI (100) |  | IL |
| 2 | 252 | 19 | -65 | -21 |  |  |  | IL |
| 2 | 252 | 13 | -57 | -16 |  | VI (78) | V (22) | IL |
|  |  |  |  |  |  |  |  |  |
| 6 | 529 | -39 | -12 | 54 | Precentral | 6 (80) | 4a (40); 1, 3b (10) | CL |
| 6 | 529 | -42 | -19 | 62 |  | 6 (70) | 1, 4a (30); 3b (20) | CL |
| 6 | 529 | -38 | -12 | 65 |  | 6 (80) |  | CL |
| 6 | 529 | -36 | -34 | 53 | Postcentral | 3b (60) | 2, 4p (40); 3a (20); SPL (7PC) (10) | CL |
| 6 | 529 | -39 | -23 | 53 |  | 3b (40) | 4p (30); 1,2, 4a (10) | CL |
| 6 | 529 | -53 | -23 | 54 |  | 1 (90) | 2 (20); 3b (10) | CL |
| 6 | 529 | -36 | -45 | 60 |  | SPL (7PC), 2 (50) | SPL (7PC), 2 (50); 1 (20); hIP3 (10) | CL |
| 6 | 529 | -49 | -15 | 55 |  | 1 (50) | 3b, 4a (20); 4p, 6 (10) | CL |
| 6 | 529 | -42 | -38 | 59 |  | 2 (50) | 1 (40); SPL (7PC), 3b (10) | CL |
| 6 | 529 | -48 | -30 | 60 |  | 1 (90) | 2 (30) | CL |
| 6 | 529 | -38 | -30 | 63 |  | 1, 4a (60) | 6 (20); 2, 3b (10) | CL |
| 7 | 75 | -2 | -5 | 55 | Supplementary Motor Area | 6 (70) |  | CL |
| 6 | 529 | -58 | -22 | 44 | Inferior parietal lobule | 1, 2 (40) | IPC (PF) (30) | CL |
| 6 | 529 | -45 | -28 | 48 |  | 2 (70) | 3b (30); 1, IPC (PFt) (20); 3a (10) | CL |
| 6 | 529 | -33 | -52 | 65 | Superior parietal lobule | SPL (7PC) (60) | SPL (7A) (40); 1,2 (20) | CL |
| 7 | 75 | 0 | -2 | 48 | Cingulum Mid | 6 (60) |  | CL |
| 6 | 529 | -59 | -23 | 36 | SupraMarginal | IPC (PFt) (70) | 2 (20); 1 (10) | CL |
| 4 | 66 | -44 | -67 | -10 | Inferior Occipital Gyrus |  |  | CL |
| 4 | 66 | -39 | -72 | -5 |  | hOC5 (V5) (20) |  | CL |
| 4 | 66 | -51 | -70 | 2 | Middle Temporal Gyrus |  |  | CL |
| 4 | 66 | -43 | -73 | 3 | Middle Occipital Gyrus | hOC5 (V5) (50) |  | CL |
| 5 | 19 | -42 | -3 | 12 | Rolandic Oper | OP 3 (10) |  | CL |
